# Reduced graphene oxide coated alginate scaffolds: potential for cardiac patch application

**DOI:** 10.1186/s40824-023-00449-9

**Published:** 2023-11-04

**Authors:** Nafiseh Baheiraei, Mehdi Razavi, Ramin Ghahremanzadeh

**Affiliations:** 1 Tissue Engineering and Applied Cell Sciences Division,Department of Anatomical Sciences, Faculty of Medical Sciences, Tarbiat Modares University, Tehran, 1411713116 Iran; 2 Department of Medicine, Biionix (Bionic Materials, Implants & Interfaces) Cluster, University of Central Florida College of Medicine, Orlando, FL 32827 USA; 3 Department of Material Sciences and Engineering, University of Central Florida, Orlando, FL 32816 USA; 4 Nanobiotechnology Research Center, Avicenna Research Institute, ACECR, Tehran, Iran

**Keywords:** Cardiac patch, Vascularization, Antibacterial, Reduced graphene oxide, Antioxidant

## Abstract

**Background:**

Cardiovascular diseases, particularly myocardial infarction (MI), are the leading cause of death worldwide and a major contributor to disability. Cardiac tissue engineering is a promising approach for preventing functional damage or improving cardiac function after MI. We aimed to introduce a novel electroactive cardiac patch based on reduced graphene oxide-coated alginate scaffolds due to the promising functional behavior of electroactive biomaterials to regulate cell proliferation, biocompatibility, and signal transition.

**Methods:**

The fabrication of novel electroactive cardiac patches based on alginate (ALG) coated with different concentrations of reduced graphene oxide (rGO) using sodium hydrosulfite is described here. The prepared scaffolds were thoroughly tested for their physicochemical properties and cytocompatibility. ALG-rGO scaffolds were also tested for their antimicrobial and antioxidant properties. Subcutaneous implantation in mice was used to evaluate the scaffolds' ability to induce angiogenesis.

**Results:**

The Young modulus of the scaffolds was increased by increasing the rGO concentration from 92 ± 4.51 kPa for ALG to 431 ± 4.89 kPa for ALG-rGO-4 (ALG coated with 0.3% w/v rGO). The scaffolds' tensile strength trended similarly. The electrical conductivity of coated scaffolds was calculated in the semi-conductive range (~ 10^−4^ S/m). Furthermore, when compared to ALG scaffolds, human umbilical vein endothelial cells (HUVECs) cultured on ALG-rGO scaffolds demonstrated improved cell viability and adhesion. Upregulation of VEGFR2 expression at both the mRNA and protein levels confirmed that rGO coating significantly boosted the angiogenic capability of ALG against HUVECs. OD620 assay and FE-SEM observation demonstrated the antibacterial properties of electroactive scaffolds against *Escherichia coli*, *Staphylococcus aureus*, and *Streptococcus pyogenes*. We also showed that the prepared samples possessed antioxidant activity using a 2,2-diphenyl-1-picrylhydrazyl (DPPH) scavenging assay and UV–vis spectroscopy. Histological evaluations confirmed the enhanced vascularization properties of coated samples after subcutaneous implantation.

**Conclusion:**

Our findings suggest that ALG-rGO is a promising scaffold for accelerating the repair of damaged heart tissue.

**Graphical Abstract:**

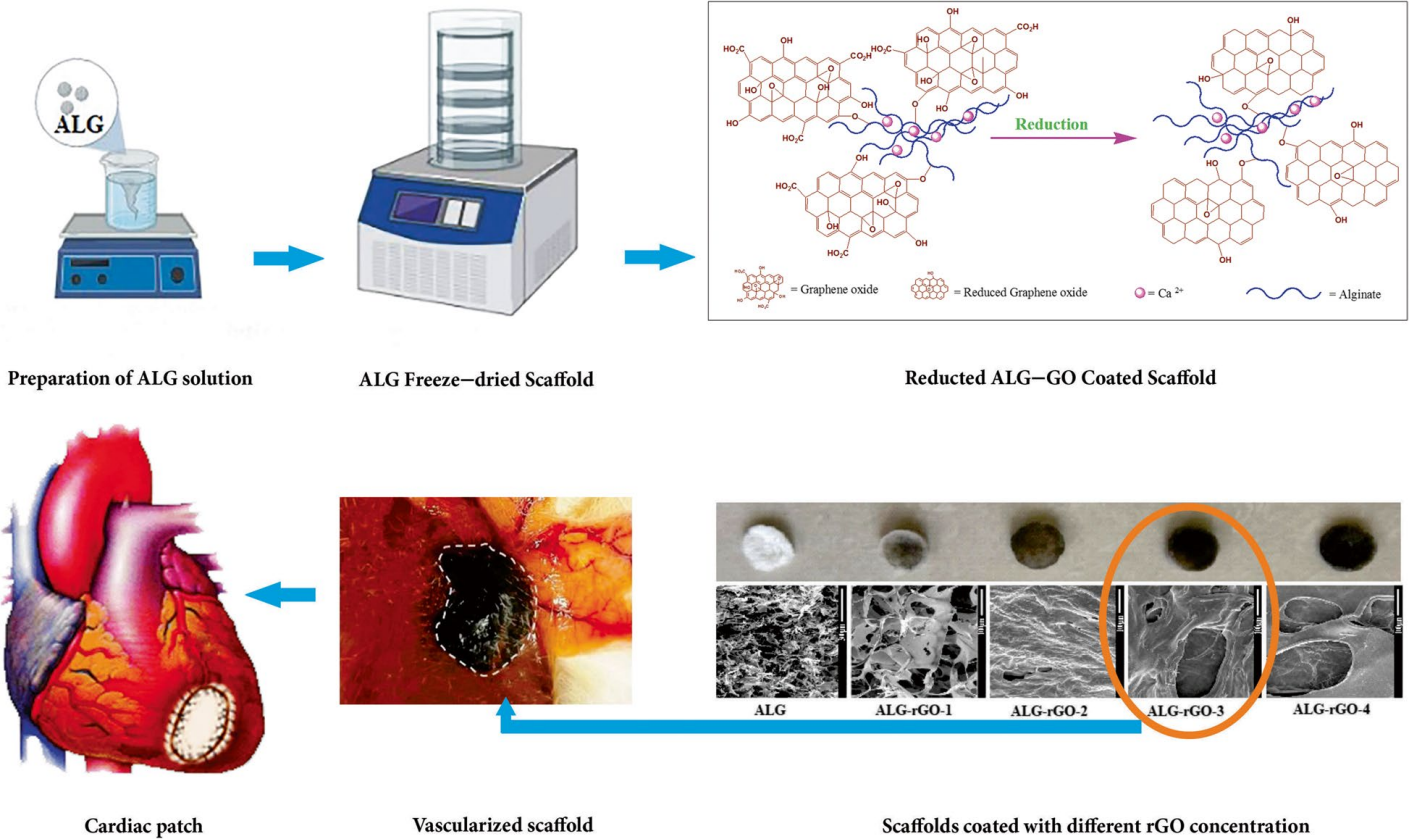

## Background

Cardiovascular diseases (CVD) cover a significant fraction of the present global diseases, constituting about 30% of all deaths globally, i.e., 19.1 million worldwide deaths annually [1]. Among CVDs, myocardial infarction (MI) is one of the leading causes of heart failure (HF), which, in turn, is the primary cause of late mortality, morbidity, and healthcare costs [2]. During MI, millions of cardiomyocytes (CMs) and vascular networks are damaged due to the ischemia that originates from occluded coronary arteries. MI causes nonfunctional remodeling, leading to left ventricular (LV) dilation, wall thinning, fibrosis, decreased cardiac function, and arrhythmia [3].

Although considerable efforts have been made to find new therapies and permanent solutions, there are still no definitive therapies for MI. The common protective and preservative treatments, such as drugs and surgeries, cannot effectively regenerate and restore cardiac tissue's mechanical and electrical aspects [4]. Despite encouraging improvements from preclinical and clinical studies of cardiac cell therapies, including reducing myocardial injury and its progression to HF, prolonged survival of transplanted cells in the cardiac microenvironment remains challenging [5] due to the low rate of cell engraftment. Heart transplantation is presently the only long-term approach for treating wide-ranging cardiac injuries. Yet, the number of donors is inadequate, and only a few people on the transplant waiting list can take on a new heart [6].

Recent attempts have been made to engineer cardiac patches as synthetic transplants to replace areas of damaged myocardial tissue. The advent of tissue engineering resulted in additional benefits of the therapeutic patch-based methods with infarcted wall strengthening, providing a template for cells to proliferate and secrete their matrix, and limiting adverse remodeling [7]. Among various materials that can be used for patch fabrication, alginate (ALG) has shown therapeutic potential for preclinical and clinical studies [8–11]. This material is promising for cardiac regeneration due to its water-solubility, biocompatibility, tunability in biodegradability, and mechanical stiffness [8]. The efficacy of intramyocardial injection of Algisyl-LVR™ -a calcium alginate hydrogel- has been previously approved for treating ischemic HF in swine by reducing wall stress and improving cardiac function [9]. This alginate- based material has also been injected for LV augmentation in patients with dilated cardiomyopathy. The feasibility and safety of treatment with this material were confirmed for patients with chronic HF [10]. Algisyl-LVR™ has been considered an inert permanent implant that, when injected into the cardiac muscle, could serve as a prosthetic scaffold to decrease wall stress and prevent further LV enlargement [11].

Since the heart is an electroactive tissue capable of electrical signal transmission, nonconductive scaffolds cannot improve the electrical integrity of an injured heart. High conductivity for cardiac patches could create electrical activity between infarcted tissue and healthy myocardium. A wide range of electroconductive biomaterials have been utilized to restore the electrical integrity of the heart. Our group [12–16] and others [17, 18] reported the satisfactory functional behavior of electroactive biomaterials to control cell proliferation, biocompatibility, and signal transition. Graphene and its derivatives, such as graphene oxide (GO) and reduced graphene oxide (rGO), have appeared as a new class of nanomaterials for numerous biomedical applications, including medical imaging, drug delivery, and tissue engineering [19]. Graphene-based materials (GBMs) have been widely used in biomedical applications due to their large number of functional groups and, particularly, their large surface-to-volume ratio, which results in unique optical, mechanical, and electrical properties. The promising characteristics of rGO incorporated within the polymeric matrix have been previously approved for cardiac tissue engineering [20] and other applications [21]. Park et al. incorporated rGO flakes into mesenchymal stem cells (MSCs) spheroids, which were then injected into the infarcted mouse heart [22]. Fibronectin-adsorbed rGO improved cell- extracellular matrix (ECM) interaction and, subsequently, expression of the paracrine factor within MSC spheroids. The authors stated that rGO could improve the therapeutic efficacy of MSCs for ischemic cardiac diseases [22].

Despite the biocompatibility of ALG as a natural polymer, the major limitation of this material in tissue engineering is the lack of interaction with mammalian cells and low protein adsorption, which does not support cell attachment [23]. Low mechanical properties and [24] a lack of electrical conductivity are other limitations that need to be assessed regarding the use of this material for cardiac repair. One possible approach to overcome this limitation is to fabricate a composite scaffold by adding reinforced materials like rGO to the ALG matrix [25, 26]. Recently, our group confirmed that intramyocardial injection of rGO/ALG hydrogel increases LV function, neovascularization, and electrical characteristics of the myocardium following MI in a model of rat chronic ischemic cardiomyopathy. It was also suggested that the presence of rGO, within ALG could deliver the desired electroactive hydrogel for stem cell therapy in patients with ischemic heart disease [27, 28].

In addition, biofilm and infection-related biomaterials should be eliminated to decrease the therapeutic risk of cardiac patch application [29]. Graphene is a green antimicrobial material that can be applied directly to wounds [30]. Thus, many researchers have described the antibacterial efficacy of GO and rGO against *Escherichia coli (E.coli)* and *Staphylococcus aureus (S.aureus)* bacteria [31–34]. Apart from the aforementioned advantages, GBMs also demonstrate noteworthy antioxidant activity in the system of hydroxyl and superoxide radical scavenging. They can protect a range of biomolecular objective molecules from oxidation [35], especially after MI, in which a significant quantity of reactive oxygen species (ROS) accumulates in the infarcted area [36]. We have also recently reviewed the effects of GBMs and their mechanism in the field of cardiac tissue engineering. More importantly, we highlighted the impact of applying these materials on angiogenesis, and discussed their antibacterial, antioxidant, electrical, and mechanical properties [37].

In this study, we aimed to benefit from the merits, as mentioned earlier, of ALG and rGO to fabricate a novel electroactive cardiac patch. ALG scaffolds were fabricated via the freeze-drying method, and then was coated with varying concentrations of GO followed by a reducing agent using sodium hydrosulfate. We were assuming that rGO could improve the antibacterial and antioxidant properties of ALG as well as the angiogenic capability of human umbilical vein endothelial cells (HUVECs) being cultured on ALG-rGO scaffolds compared to the non-modified ALG.

## Materials & methods

### GO synthesis

In this study, GO nanosheets were produced based on the Hummers method with slight modifications [38]. Briefly, 1 g of graphite powder (Merck) and 1 g of sodium nitrate (NaNO_3_, Sigma-Aldrich) were poured into a 250 ml round -bottom flask and kept for 10 min. in an ice bath. Next, 50 mL of concentrated sulfuric acid (H_2_SO_4_, 96%, Merck) was added to the mixture and stirred while homogenizing for about 20 min. The temperature was controlled to around 10 °C while adding 7 g of potassium permanganate (KMnO4, Merck) gradually to the reaction flask for 2 h. The reaction flask was then placed in a water bath of 35 °C and stirred for 2 h. Next, the bath temperature was set to 95 °C, and 90 ml of deionized (DI) water was gently added to the reaction mixture. Then, 45 ml of DI water was added to the flask simultaneously, followed by adding 3 ml of hydrogen peroxide (H_2_O_2_, 30%, Sigma-Aldrich) dropwise into the reaction system, resulting in the formation of a yellowish-brown suspension. The formed solid powders were centrifuged at 8000 rpm for 8 min, and the obtained precipitation was washed three times using diluted hydrochloric acid (HCl, 3%, Merck) and DI water. Exfoliation of graphite oxide was performed by sonication in DI water at room temperature for 1 h, resulting in uniform GO dispersion. Finally, the frozen product (at -80 °C for 10 h) was placed in a freeze-dryer (Alpha 1–4 LDplus, Martin Christ, Germany) for 24 h. The formed GO powder was stored in a vacuum desiccator for experiments.

### Scaffold synthesis

ALG scaffolds were fabricated via a conventional freeze-drying method. First, an aqueous solution of sodium alginate (2% w/v; Sigma-Aldrich) was prepared and cast in Teflon molds and then placed in a freezer with a temperature of -20 °C for 8 h, followed by 12 h at -80 °C. Samples were next lyophilized in a freeze-dryer device (Alpha 1–4 LD plus, Martin Christ, Germany) for 48 h at -80 °C. The scaffolds were cross-linked by immersion in calcium chloride solution (CaCl_2_; Sigma-Aldrich) at 4 °C for 4 h and then freeze-dried again for a further 24 h.

To coat the ALG scaffolds, an aqueous homogenous GO solution treated by a probe sonicator device (Q700, Thomas Scientific) was made with different concentrations (0.05%, 0.1%, 0.2%, and 0.3% w/v). Two hundred microliters of the prepared GO solution were then injected into the ALG scaffolds (1 × 1 cm), followed by a reduction in 2% w/v sodium hydrosulfite (Na_2_S_2_O_4_, Sigma-Aldrich) for 3 min. Samples were then put at 70 °C for 1 h and later washed well with ethanol and air dried [39]. The prepared scaffolds were finally labeled, as shown in Table 1.

**Table 1:** Scaffold preparations and groups

Abbreviations	Scaffolds
ALG	Pristine Alginate
ALG-rGO-1	Alginate coated with 0.05% w/v (5 mg/ml)GO followed by reduction
ALG-rGO-2	Alginate coated with 0.1% w/v (10 mg/ml) GO followed by reduction
ALG-rGO-3	Alginate coated with 0.2% w/v (20 mg/ml) GO followed by reduction
ALG-rGO-4	Alginate coated with 0.3% w/v (30 mg/ml) GO followed by reduction

### GO characterizations

Fourier transform infrared (FTIR) spectra were performed using Equinox-55 (Bruker, Madison, Wisconsin, USA) in the 4000–400 cm^−1^. The morphology and dimension of the graphene oxide nanosheets were studied by atomic force microscopy (AFM; Dualscope/Rasterscope C26, DME, Denmark). Structural studies and compositional features of the flakes were also evaluated using an X-ray diffractometer (XRD; Rigaku D/max-3C, Japan).

### Scaffold characterizations

#### Physicochemical characterizations

The chemical composition and bonds of the scaffolds were evaluated using Attenuated Total Reflection-Fourier Transform Infrared Spectroscopy (ATR-FTIR; AVATAR, Thermo, USA). All spectra were documented in the 400–4000 cm^−1^ range at a scan speed of 64 scans/min with a resolution of 8 cm^−1^ in a KBr-diluted medium. The structural phases of the fabricated samples were analyzed using XRD (EQUINOX3000, Inel, France) with a Cu anode at a fixed incident angle of 0.03 in a 2θ range of 5–105°. For these two tests, ALG-rGO-1 was used to represent electroactive scaffolds. The morphology, microstructure, and cross-section of the scaffolds were also examined by scanning electron microscopy (SEM; Tescan Vega II, Czech) after gold coating. The porosity of the scaffolds was calculated according to Archimedes’s liquid displacement principle using the following formula:

1 $$\mathrm{Porosity }(\mathrm{\%}) =\frac{(\mathrm{Wt }-\mathrm{Wr}-\mathrm{Wd})}{(\mathrm{Wc }-\mathrm{Wr})} \times 100$$

where W_c_ is the weight of the container filled with ethanol, W_d_ is the weight of the dry scaffold, W_t_ is the total weight of the scaffold along with the container after being soaked in ethanol, and W_r_ is the remaining mass after removing the scaffold [40]. The pore size of the scaffolds was calculated using Image J software (National Institutes of Health, Bethesda, USA). The surface hydrophilicity of the scaffolds was assessed by water contact angle measurement via an optical video contact angle system (CAG-20, Jikan, Iran) using the sessile drop method. After dropping 4 μl of water over the surface of the scaffolds, changes in the water droplet shape were recorded, and the surface contact angles were calculated. The average value of at least three different spots was considered for each scaffold. To evaluate the hydrolytic degradation of the scaffolds, the weight of dried and sterilized samples was measured before being immersed in phosphate-buffered saline (PBS, pH = 7.4) at 37 °C. Samples were removed, washed with DI, and dried in a vacuum at specific time points. The following formula calculates the weight loss percentage:

2 $$\mathrm{Weight\,loss }\left(\mathrm{\%}\right)=\frac{\left(\mathrm{W}1-\mathrm{W}2\right)}{\mathrm{W}1}\times 100$$

where W_1_ and W_2_ are denoted by the weights of the scaffold before and after degradation, respectively.

The scaffold’s electrical conductivity was measured using a conventional four-point probe Keithley instrument (Model 196 System DMM, USA) at ambient temperature according to the following formula:

3 $$\upsigma\,(\mathrm{S\,}/\mathrm{\,m})\,=(2.44 \times\,\frac{10}{\mathrm{S}}\,\times\,(\frac{\mathrm{I}}{\mathrm{E}})$$

where σ is the conductivity in Siemens per meter (S/m), S is the sample side area in m^2^, I is the current passing the outer probes in amp, and E is the voltage drop across the inner probe in V. Also, 2.44 is referred to as the systematic constant [15].

The mechanical properties of the samples were evaluated using a tensile testing machine (ZwickRoell Z050, Germany). Scaffolds were carefully cut into rectangular strips (1 × 3 cm) and stretched at an extension rate of 10 mm/min till rupture. The tensile strength and Young’s modulus were calculated from the linear region of a stress–strain curve. The test was repeated three times for each sample.

#### Cytotoxicity and morphological assessments

The cytotoxicity of the prepared scaffolds was examined using human umbilical vein endothelial cells (HUVECs, National Cell Bank, Pasteur Institute, Iran). Cells were cultured in a medium composed of Dulbecco’s Modified Eagle Medium F12 (DMEM F12; Invitrogen) added with 1% antibiotic penicillin/streptomycin (Sigma-Aldrich) and 10% (v/v) fetal bovine serum (FBS; Gibco). The culture medium was changed every other day, and cells were split after reaching 80%–90% confluency. Passage-3 cells were used for this experiment. Before cell seeding, samples were sterilized with 70% ethanol (Zakaria Jahrom), and washed in DI water. Cell biocompatibility was assessed using the 3-(4,5dimethylthiazol-2-yl)-2,5-diphenyltetra-zoliumbromide) (MTT; Sigma) colorimetric assay. For this evaluation, 3 × 10^3^ cells were seeded over scaffolds in a 96-well plate and incubated under standard culturing conditions for 48 and 72 h. At each time point, the medium was discarded, and 100-μL MTT solution (5 mg/mL in PBS) was added to each well. After four hours of incubation, the medium was removed, and the formazone precipitates were dissolved in dimethyl sulfoxide (DMSO; Sigma-Aldrich). The optical absorbance at 570 nm was recorded using a microplate Elisa reader (ELX808, BioTek, USA). Cells cultured on a tissue culture plate (TCP) were considered the control group. SEM was also used to analyze the morphology of cells cultured on scaffolds. For this study, at 48 h post- seeding, samples were rinsed with PBS, and cells were fixed with 2.5% glutaraldehyde (Sigma-Aldrich) for 1 h at room temperature. Dehydration was then performed using graded ethanol series before being coated with gold for SEM observation.

#### Angiogenesis assays in vitro

The capability of the prepared scaffolds to induce upregulation of expression of Vascular Endothelial Growth Factor Receptor 2 (VEGFR2) was evaluated at both mRNA and protein levels against HUVECs. The obtained data of HUVECs seeded on scaffolds were compared with cells on TCP.

#### RNA extraction and real-time reverse transcription PCR (qRT-PCR)

The gene expression of VEGFR2 for HUVECs seeded on the scaffolds was investigated after 7 days by quantitative reverse transcription PCR (qRT-PCR). Total RNAs were extracted using the RiboEX kit (GeneALL biotechnology, Korea) as reported by the manufacturer’s instructions. Genomic DNA removal and cDNA synthesis were carried out utilizing DNase I, RNase-free kit (Takara, Japan), and Easy cDNA Synthesis Kit (Parstous, Iran).

QRT-PCR was performed using the RealQ Plus 2 × Master Mix Green (Ampliqon, cat.no. A323402, Denmark) by the Rotor Gene6000 Real-Time PCR System (QIAGEN, Germany). Nearly 5 µL of the synthesized cDNA and 0.5 µL of each primer in a total of 20 µL were used for the PCR reaction step. For relative gene expression analysis, the Pfaffl method was used, and all cycle threshold (*Ct*) values were calculated from the target gene (VEGFR2), which were normalized to internal control) GAPDH (. Cells cultured on TCP were considered the control group.

#### Immunohistology staining

Immunofluorescence staining was conducted for comparative evaluation of protein expression of VEGFR2 for HUVECs seeded on the scaffolds after 7 and 14 days. Briefly, culture media were removed at each time point, scaffolds were washed with PBS, and cells were fixed with 4% paraformaldehyde (Sigma-Aldrich) for 30 min. at room temperature. Permeabilization was performed with 0.2% saponin in a blocking buffer (10% goat serum in PBS) for 15 min at room temperature. Scaffolds and cells were incubated with a primary antibody of anti-VEGFR2 (Abcam, USA, 1:100) at 4 °C overnight. Subsequently, samples were incubated with the secondary antibody (goat anti-rabbit IgG conjugated with Alexa Fluor® 488, ab150077, USA, 1:1000) for 1 h at 37 °C. Nucleus staining was made using 4’,6- Diamidino-2-Phenylindole (DAPI, Sigma). Cells were observed using a fluorescence microscope (Olympus BX51, Japan). Negative controls were performed by incubating only secondary antibody. The protein expression ratio was quantified using Fiji/ImageJ (version 1.8.0., LOCI, University of Wisconsin, USA) analysis software.

#### Antimicrobial and antioxidant evaluations

*Escherichia coli* (ATCC 25922), *Staphylococcus aureus* (ATCC 25923), and clinically isolated *Streptococcus pyogenes* were prepared from the archive of the Pasteur Institute of Iran. For the antimicrobial assay, bacteria were cultured at 37 °C in the brain–heart infusion (BHI) broth medium (Difico Co.) and kept in a shaking incubator to reach an optical density (OD620 nm) of 0.13 equivalent to 1.5 × 10^8^ CFU/ml, which were then spread-cultured on BHI agar medium. The sterilized samples were placed on the plate for 24 h before being fixed with 2.5% glutaraldehyde (Sigma-Aldrich) for 1 h. at room temperature. Scaffolds were then dehydrated using serial alcohol solutions of 40–100%, all for 15 min, for field emission scanning electron microscopy (FE-SEM, Mira 3-XMU, Czechia) observation.

Also, for the OD620 assay, bacterial cells were cultured in 96 microwell plates over the scaffolds. After 6 h incubation, 100 μl of the bacterial suspensions were removed to a new plate, and absorption was measured at 620 nm using a microplate reader [41]. The concentration of each bacterial suspension in the presence of the samples was calculated and compared with the positive control containing the Müller-Hinton broth and bacterial suspension. For the negative control, only the Müller-Hinton broth was used.

The ability of rGO-coated scaffolds to scavenge ·OH was investigated using 2, 2-diphenyl-1-picrylhydrazyl (DPPH, Sigma-Aldrich) free radical assay in which DPPH• concentration was observed using a UV–Visible spectrophotometer. Briefly, 0.35 mM DPPH solution was prepared with methanol, and 2 mg of the scaffolds was added to the solution. This mixture was next vortexed and incubated for 20 min. in the dark. The absorbance of the treated scaffolds (Abs _scaffold_) was measured using a UV–Visible spectrophotometer at 517 nm [42]. The absorbance of the untreated DPPH solution (Abs _DPPH_) was also considered as control. The antioxidant activity of the samples was quantified as follows:

4 $$\mathrm{Antioxidant\,activity\,}(\mathrm{\%})\,=\frac{(\mathrm{Abs\,DPPH}-\mathrm{Abs\,scaffold})}{\mathrm{Abs\,DPPH}}*100$$

#### In vivo assessments and immunohistochemistry staining

The angiogenesis-inducing capability of the scaffolds was assessed by subcutaneous implantation of the scaffolds after 1 and 3 weeks. Animal experiments were performed according to the ethics committee guidelines for laboratory animals approved by the Ethics Committee of Tarbiat Modares University, Iran (IR.MODARES.AEC.1401.054). Eighteen male NMRI mice (25–30 g, aged 7 weeks) received cyclosporine (Novartis Pharma AG, Switzerland) with their drinking water 3 days before transplantation. Anesthesia was performed via intraperitoneal injection of ketamine (Alfasan, The Netherlands) and xylazine (Alfasan, The Netherlands). A transverse incision (approximately 2 cm in length) was made on the back of the mice, and the sterilized scaffolds (10 × 10 mm^2^) were subcutaneously implanted, followed by closing the incision with a surgical suture. After the mice recovered from the surgery, they had access to food and water. Vascularization was observed near and inside the scaffolds using a stereo microscope (SMZ 1000, Japan). After explanting the implantation bed, tissue was fixed in 10% buffered formalin for 24 h, then transferred into PBS at 4 °C. Fixed tissues embedded in paraffin were sectioned into several segments of 5 µm thickness and stained with Mayer’s hematoxylin and eosin (H&E; Sigma). The rate of cell migration within scaffolds was calculated as follows:

5 $$\mathrm{Cell\,migration\,rate}=\,\left(\frac{\mathrm{w}}{\mathrm{W}0}\right)\mathrm{\%}$$

where W is the area of cell migration into the scaffold, and W_0_ is the area of the scaffold [43].

To assess neovascularization, immunohistochemistry (IHC) was carried out for the endothelial cell markers CD31 and alpha-smooth muscle actin (α-SMA). Paraffin-embedded tissues were used with Anti- α-SMA (Abcam, ab5694) and Anti-CD31 (Abcam, ab28364). For this experiment, the 4-μm paraffin sections were placed on poly-L-Lysine coated slides. The inhibition of endogenous peroxidase was achieved by incubating with 10% hydrogen peroxidase in PBS for 10 min after being deparaffinized. For heat antigen retrieval, the tissue sections were heated at 95 °C with sodium citrate buffer (10 Mm, pH = 6) for 30 min. After washing with PBS, sections were incubated with primary antibodies at 4 °C overnight. The slides were then washed with PBS and incubated with the secondary antibodies at 25 °C overnight for 50 min. 3,3′-Diaminobenzidine (DAB) was utilized as a chromogen and counterstaining was performed using Meyer’s hematoxylin for 5 min [44].

Micro Vessel Density (MVD) measurement was calculated on slides stained with CD31. Immunoreactivity of the endothelial cells was considered in hot spot areas. The number of vascular lumens was counted per 4 high-power fields (HPF) and the sum was reported as the MVD of the samples. For α-SMA evaluation, the modified Allred scoring method was used, considering the proportion (P) and intensity (I) scores, as has been previously studied [45]. The two scores were added together for a final score (P + I) with eight possible values (0–1 = negative, 2–3 = mild, 4–6 = moderate, and 7–8 = strongly positive). Table 4 shows how the respective proportion and intensity scores are derived.

Photomicrographs were taken with an Olympus microscope (Olympus CH-2) and images were further quantified by imageJ software (1.53t version, USA). The results were expressed as the number of microvessels.

### Statistical analysis

Statistical analyses were performed by two-way ANOVA and multi-comparison post-hoc Tukey’s test was used to determine intra-group differences with respect to time. Graph Pad Prism software (version 8.1.2) was used for the calculations. Results were expressed as mean ± SEM. The differences with *P*-values of < 0.05 were considered statistically significant. At least three samples were tested for each experiment.

## Results

### GO flake characteristics

**Fig. 1:**
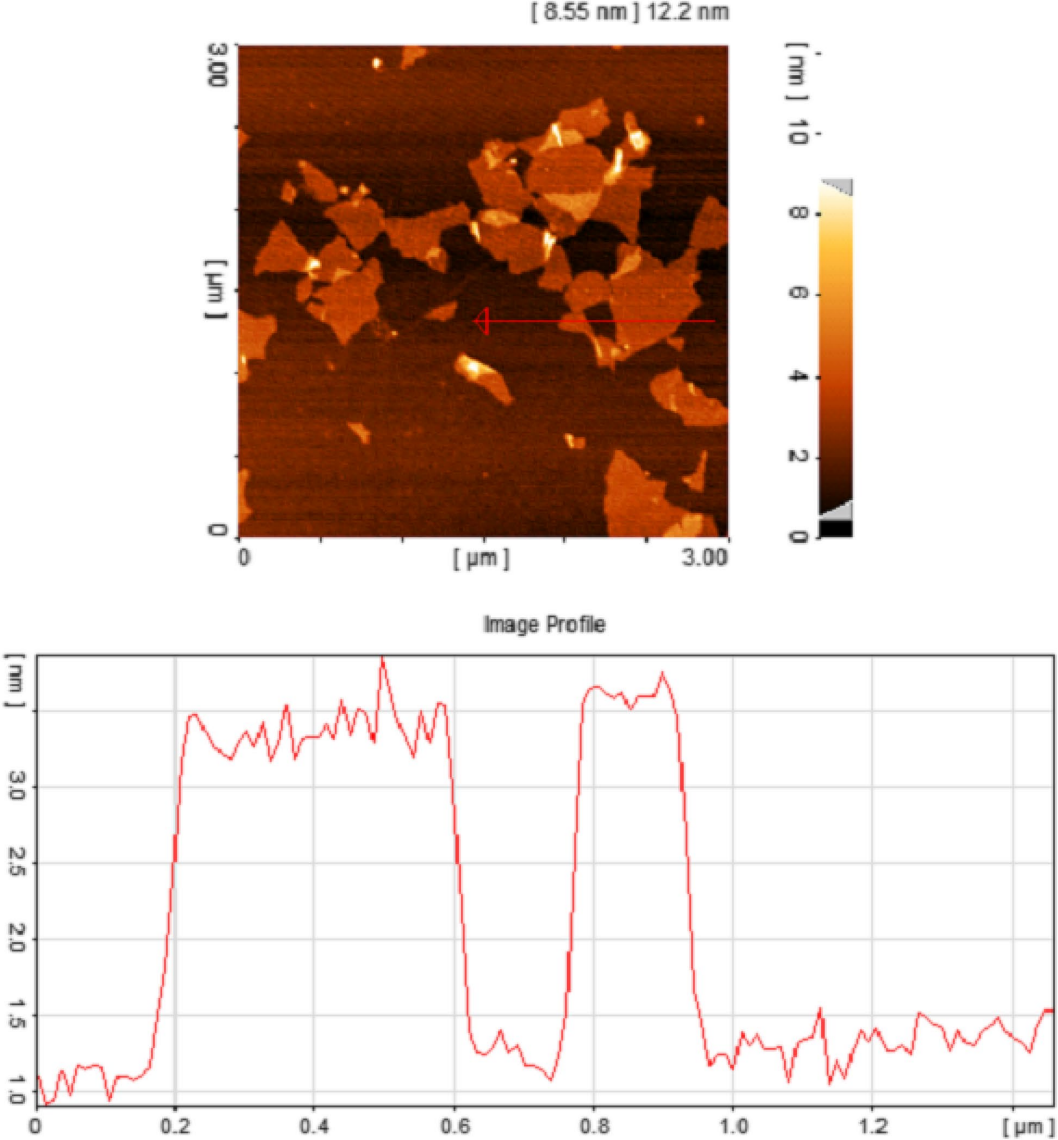
AFM image of GO flakes and height profiles of GO nano sheets

**Fig. 2:**
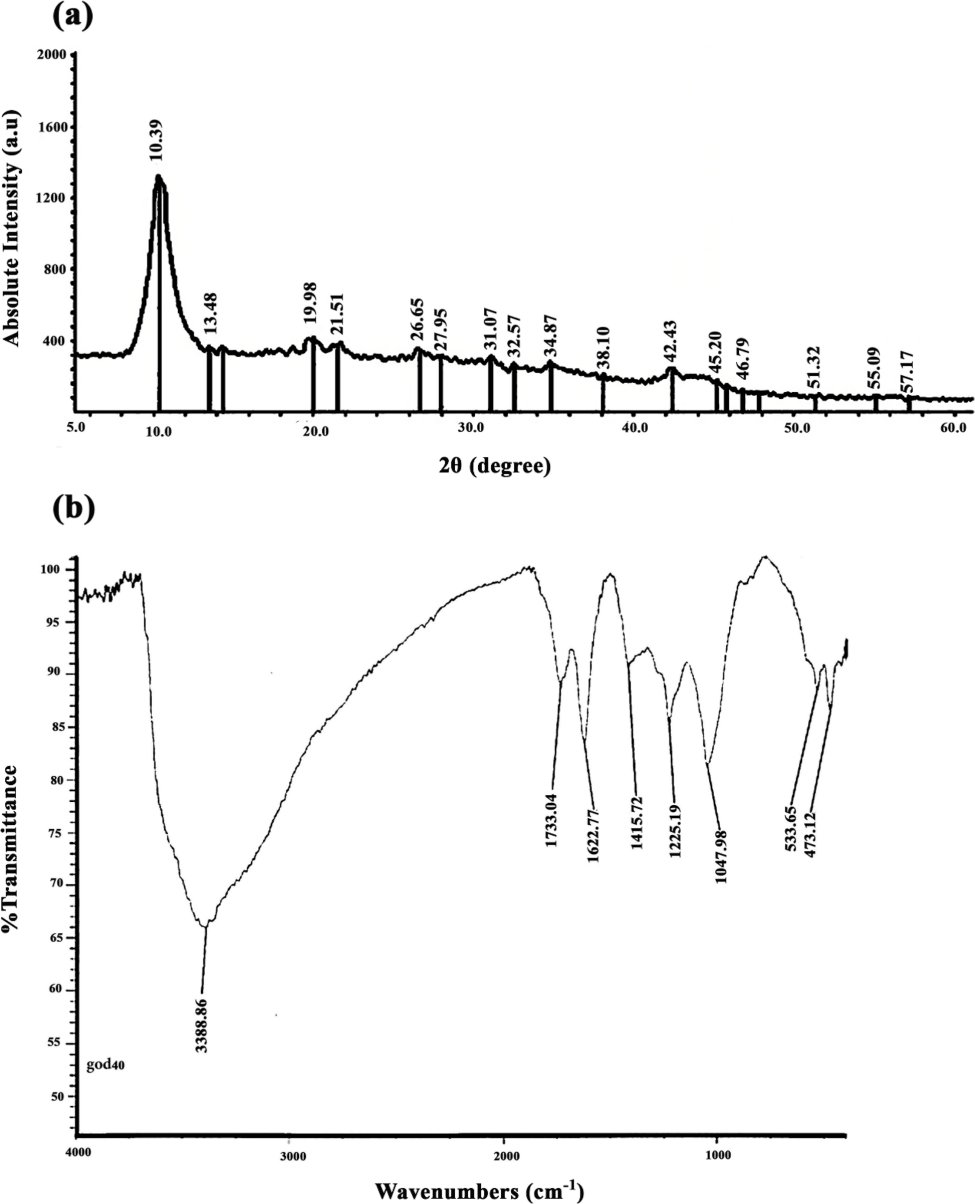
(**a**) X-Ray diffraction pattern of GO Nano sheets, (**b**) FTIR spectrum of GO nano sheets

A variety of oxygen-containing groups in the GO sheet surface make it particularly prone to interact with other sets to complete its functionalization or hybridization with additional supplementary ingredients. In this study, we measured the thickness and size of the synthesized GO sheet by AFM. The results demonstrated a thickness of ~ 2 nm with a lateral dimension of around 200–800 nm for synthesized flakes indicating that the flakes are monolayers (Fig. 1). X-Ray Diffraction pattern showed a sharp peak at 2θ = 10.39°, which is related to the (001) diffraction peak of disordered GO [46] (Fig. 2a). To confirm the successful synthesis of GO, FTIR spectral analysis was carried out. This spectrum showed the appearance of characteristic peaks for the C = O carbonyl stretching at 1733 cm^−1^, the C–OH stretching at 1225 cm^−1^, and peaks at 1047 cm^−1^ and 1415 cm^−1^, which are the characteristics peaks for C–O–C and C–OH stretching vibration frequency of GO [47], respectively. The spectrum also showed a C = C peak at 1620 cm^−1^ corresponding to the remaining sp^2^ character and a broad intense peak at 3388 cm^−1^ for OH stretching frequencies. These functional groups cause GO to be highly hydrophilic and dispersible [48] (Fig. 2b).

### Scaffold characteristics

**Fig. 3:**
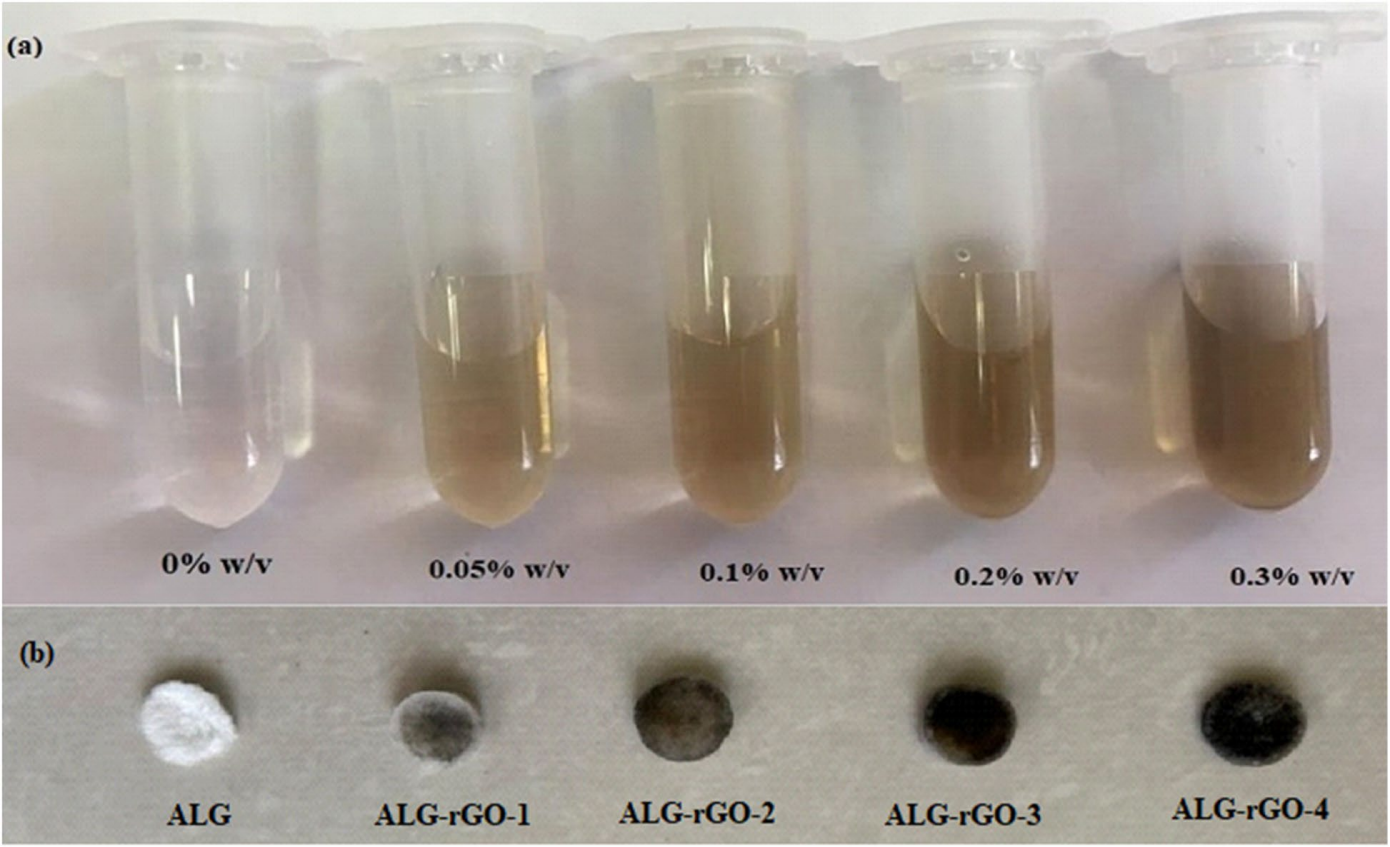
Macroscopic images of (**a**) aqueous GO solutions with different concentrations (**b**), and fabricated scaffolds

Fabricated scaffolds were assigned, as shown in Table 1. Also, the aqueous GO solutions with different concentrations and the macroscopic images of the prepared scaffolds are presented in Fig. 3. The color of the scaffolds darkened as the concentration of GO flakes was enhanced.

**Fig. 4:**
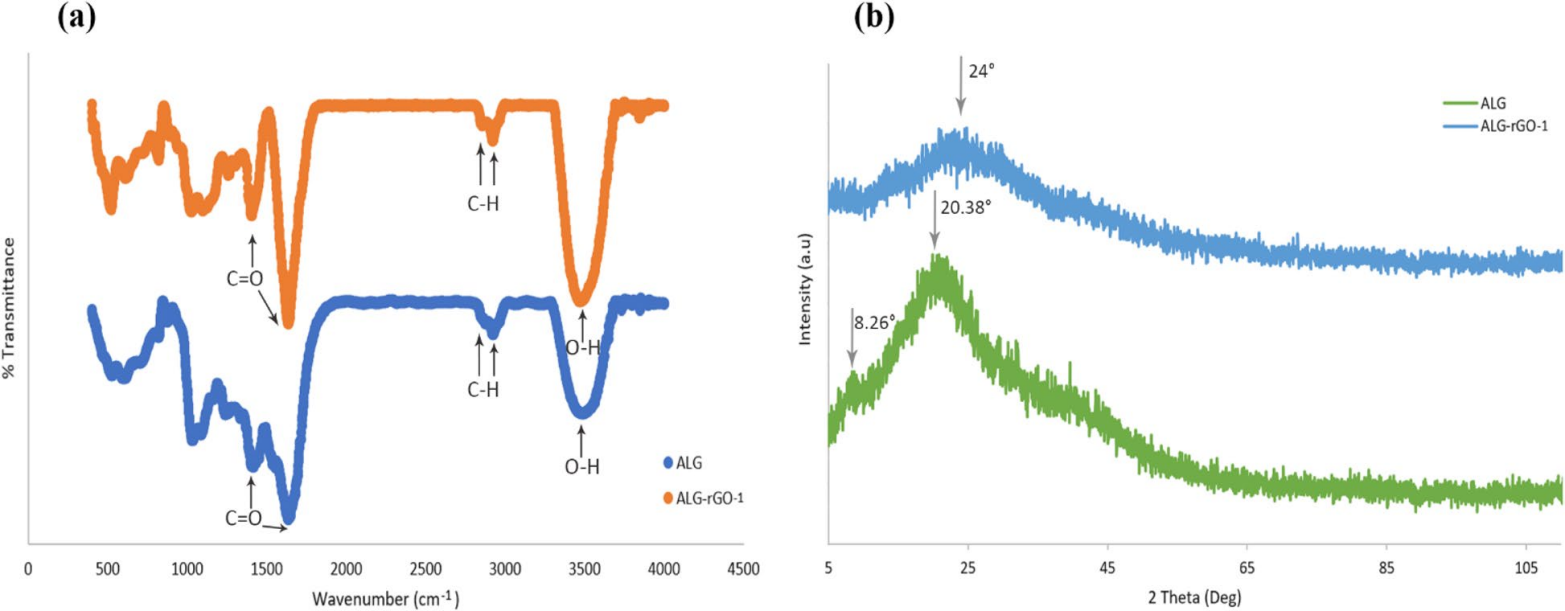
(**a**) FTIR patterns of the prepared scaffolds, (**b**) XRD patterns of the prepared scaffolds

FTIR analysis results of the scaffolds are shown in Fig. 4a. In ALG spectrum, a relatively broad peak and two sharp peaks are seen at 3494, 1633, and 1403 cm^−1^, which could be attributed to O–H stretching, C = O asymmetric, and C = O symmetric stretching vibrations of ALG, respectively. Moreover, the peaks of asymmetric and symmetric vibrations of C-H stretching can be observed at 2923 and 2854 cm^−1^ [49]. In the spectrum of ALG-rGO, the peaks of O–H stretching at 3496 cm^−1^, C = O asymmetric and symmetric stretching at 1633 and 1415 cm^−1^, and C-H asymmetric and symmetric stretching at 2929 and 2867 cm^−1^ are associated with both ALG and rGO. The indicated peak at 1633 cm^−1^ has become more intense due to overlapping the peaks of asymmetric C = O and C = C stretching and the rGO presence. Also, the O–H bonding of both ALG and rGO has led to a sharper peak at 3496 cm^−1^ compared to the spectrum of ALG [6, 27].

Based on the XRD patterns of ALG and ALG-rGO-1 scaffolds represented in Fig. 4b, ALG scaffolds exhibited two peaks at 8.26° and 20.38°, which demonstrate the existing mannuronic and guluronic acid structures in this scaffold. The obtained results were in accordance with previous investigations [50, 51]. Also, a moderately sharp peak observed at 24° in the pattern of the ALG-rGO-1 sample, which is attributed to the diffraction from the (002) plane and indicates the layered graphite structure related to the diffraction of the graphite phase [52].

**Fig. 5:**
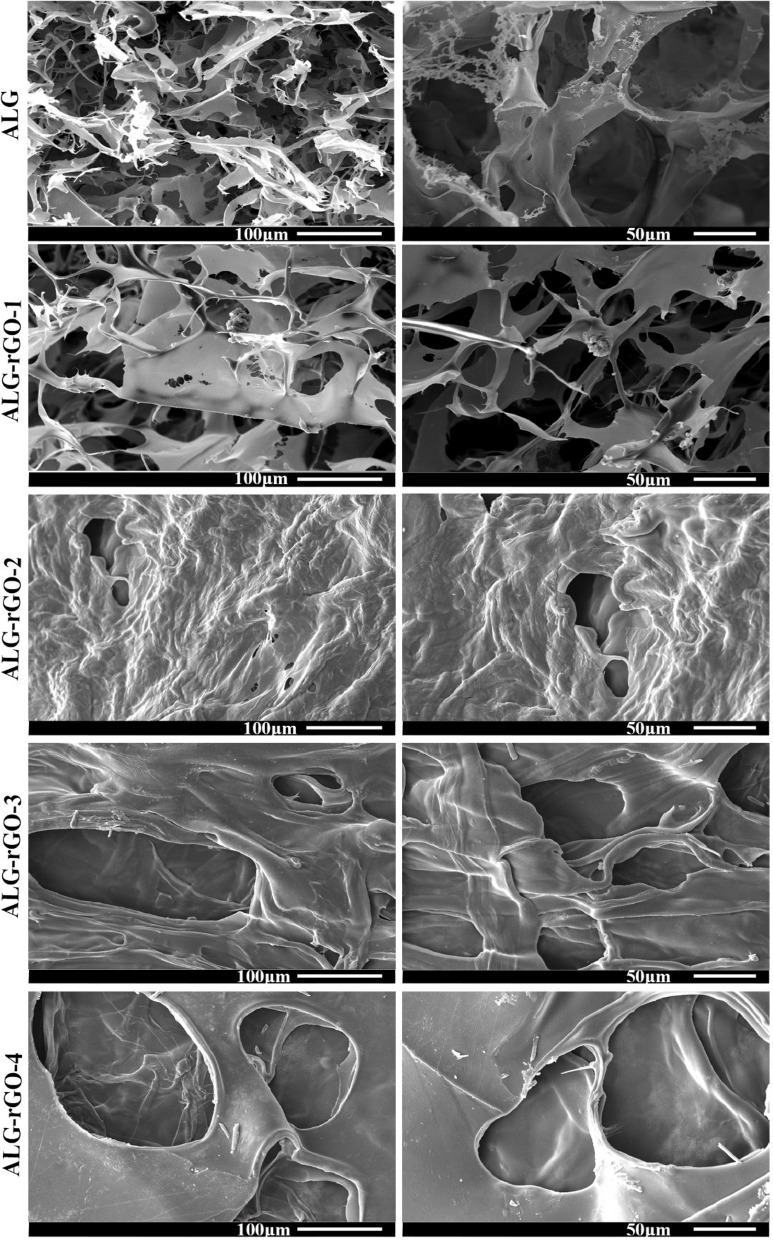
SEM images of the prepared scaffolds with different magnifications

**Fig. 6:**
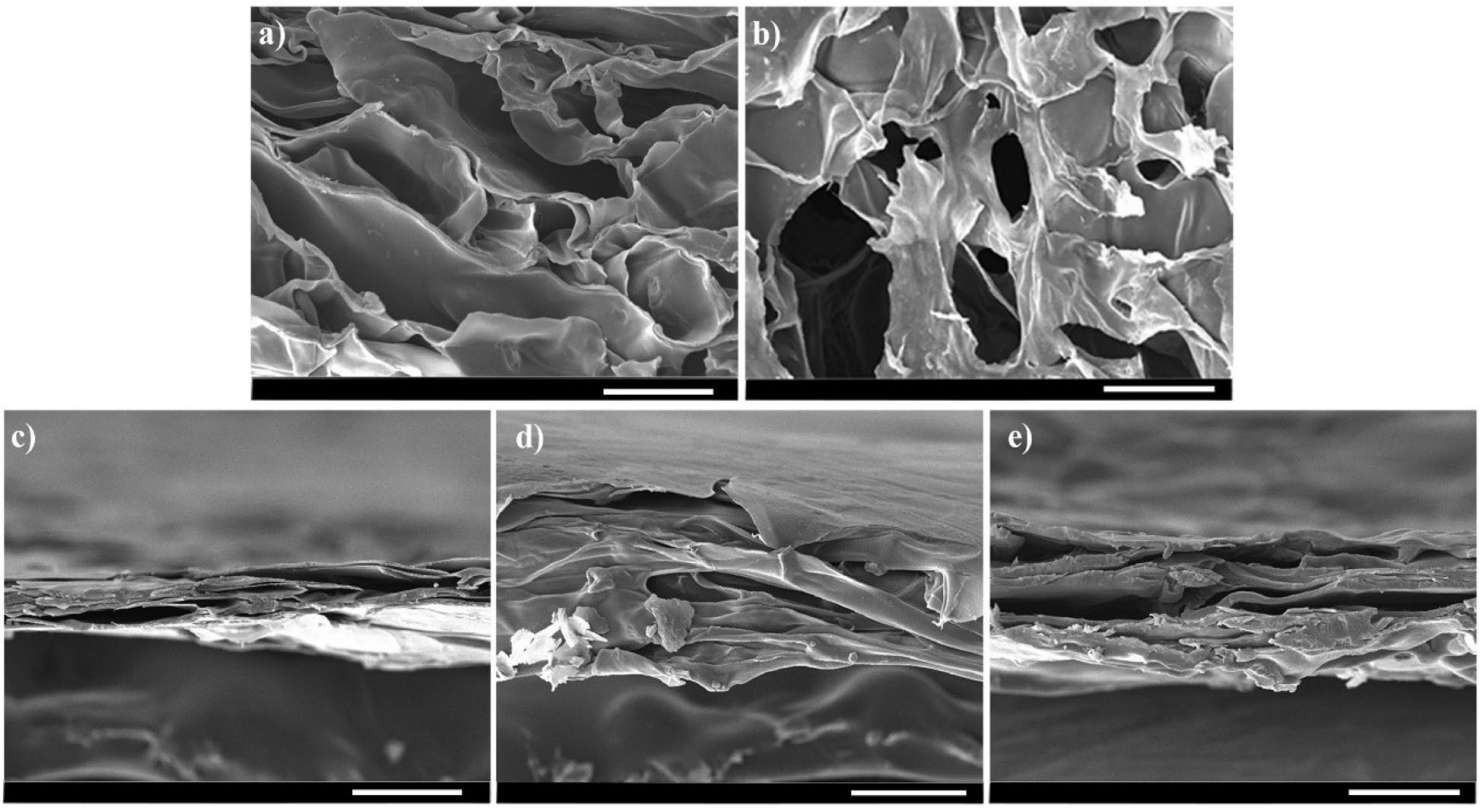
SEM images of the cross-sectionof the prepared scaffolds with different magnifications. **a**) ALG, **b**) ALG-rGO-1, **c**) ALG-rGO-2, **d**) ALG-rGO-3, **e**) ALG-rGO-4. Scale bar = 100 μm

SEM images of the prepared scaffolds revealed a porous structure for all samples (Fig. 5). While the coating had an effect on the pore arrangement, the porous configuration was preserved. For further investigations, images were also taken from the cross-section view. All scaffolds retained their porous, interconnected structure following coating and reduction (Fig. 6).

The physicochemical properties of the scaffolds are summarized in Table 2. While ALG presented the approximate pore diameters at 120.99 ± 14.08 μm, the average pore size of other scaffolds decreased as rGO concentration increased. It ranged from 108.23 ± 16.81 μm for ALG-rGO-1 to about 56.36 ± 3.71 μm for ALG-rGO-4. Apart from ALG, with about 72.5% porosity, an increase in GO concentration changed the average porosity of the coated scaffolds, reaching almost half of the value (32.66%) for ALG-rGO-4. The reduction in porosity was also visible in SEM images. A significant feature that influences the interaction between the cells and the surface of the scaffolds is hydrophilicity which was investigated by water contact angle assessment. As expected, the contact angle of the scaffolds increased by increasing the concentration of rGO from 52.3° for ALG to 71.83° for ALG-rGO-4. The electroactivity of the scaffolds, as the main element for cardiac tissue engineering, was examined via a four-point probe examination. The acquired results, which are presented in Table 2, followed an increasing trend by adding rGO concentration, reaching a value of 9.15 × 10^−4^S/m for ALG-rGO-4. The Young’s modulus of the scaffolds was determined to assess the effect of different concentrations of rGO coating on the ALG matrix on mechanical properties. The modulus was measured from the linear region of the stress–strain curve. As shown in Table 2, ALG showed the lowest stiffness with a young modulus of 92 kPa. The Young’s modulus was increased to about 150, 215, 232, and 431 kPa for rGO concentrations of 0.05, 0.1, 0.2, and 0.3% w/v, respectively. Also, by increasing the rGO concentration, the tensile strength of the scaffolds was increased, ranging from 110 kPa for ALG-rGO-1 to 200 kPa for ALG-rGO-4.

**Table 2:** Physico-chemical characteristics of the prepared samples (*n* = 3)

Samples	ALG	ALG-rGO-1	ALG-rGO-2	ALG-rGO-3	ALG-rGO-4
Pore size (μm)	120.99 ± 14.08	108.23 ± 16.81	83.97 ± 22.91	79.54 ± 27.42	56.36 ± 33.71
Porosity (%)	72.5 ± 1.66	66.66 ± 0.01	50.55 ± 8.05	46.6 ± 4.44	32.66 ± 3.83
Contact angle (°)	52.3 ± 2.6	58.45 ± 0.95	59.55 ± 1.45	60.1 ± 0.1	71.83 ± 5.11
Conductivity (S/m)	3.78 × 10^–6^	2.45 × 10^–5^	2.10 × 10^–4^	3.83 × 10^–4^	9.15 × 10^–4^
Young’s modulus(kPa)	92 ± 4.51	150 ± 5.23	215 ± 5.21	232 ± 6.32	431 ± 4.89
Tensile strength(kPa)	40 ± 4.71	110 ± 2.42	120 ± 3.81	140 ± 3.75	200 ± 2.43

**Fig. 7:**
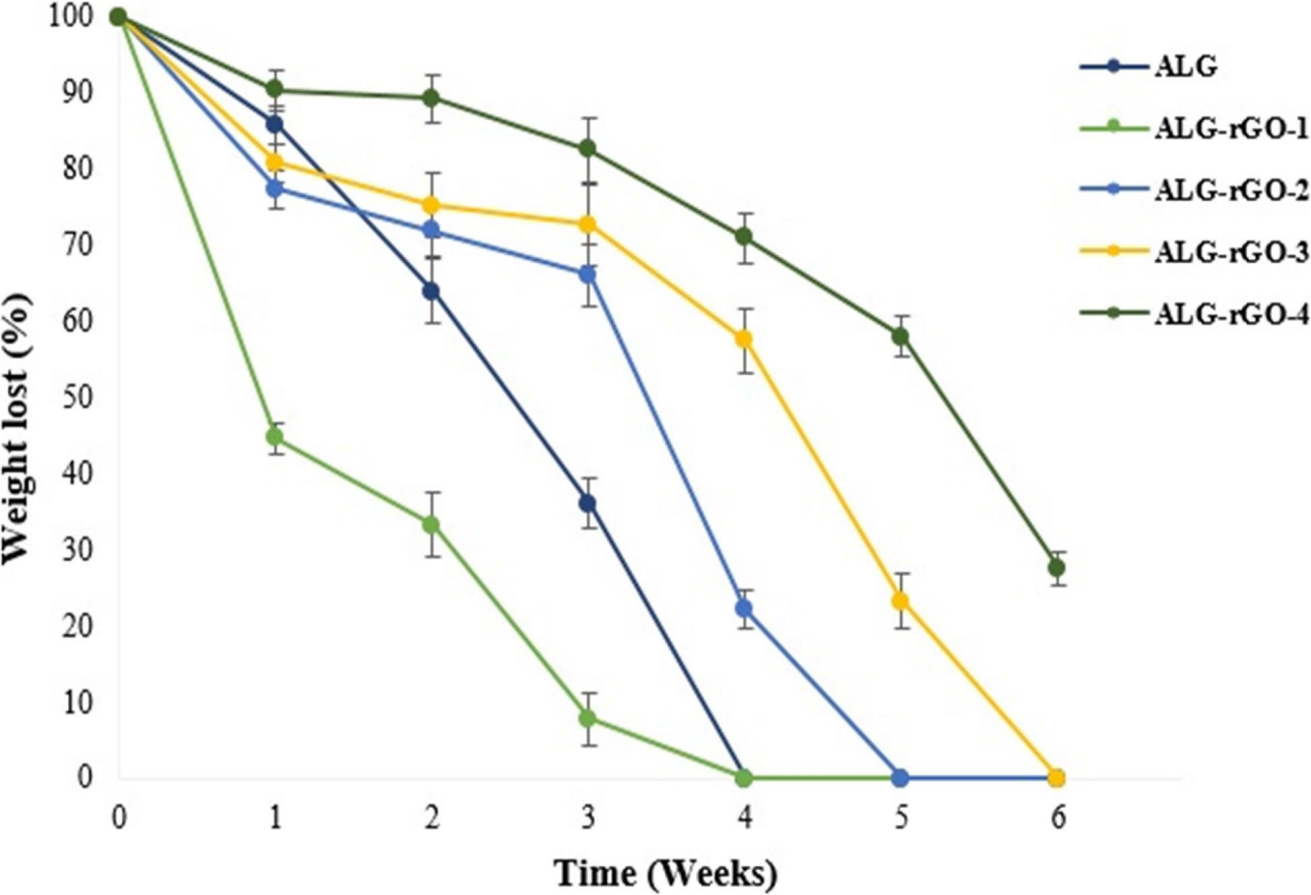
Degradation rate of prepared scaffolds in PBS (pH 7.4). The result is presented as the mean ± SD for *n* = 3

Figure 7 shows the weight loss percentage for prepared scaffolds over 6 weeks. As can be observed, all scaffolds experienced weight loss during the experimental period. ALG and ALG-rGO-1 samples were rapidly degraded in the first week; both lost almost their whole structure at the end of the fourth week. However, other rGO-coated scaffolds experienced more stable weight loss, which was influenced by the rGO concentration. While ALG-rGO-3 lost its total weight in 6 weeks, ALG-rGO -4 encountered a gradual decrease in weight loss, losing approximately 72.45% of its weight after 6 weeks. This further confirmed the higher hydrophobicity of this scaffold due to the higher rGO addition.

### Cellular evaluations

**Fig. 8:**
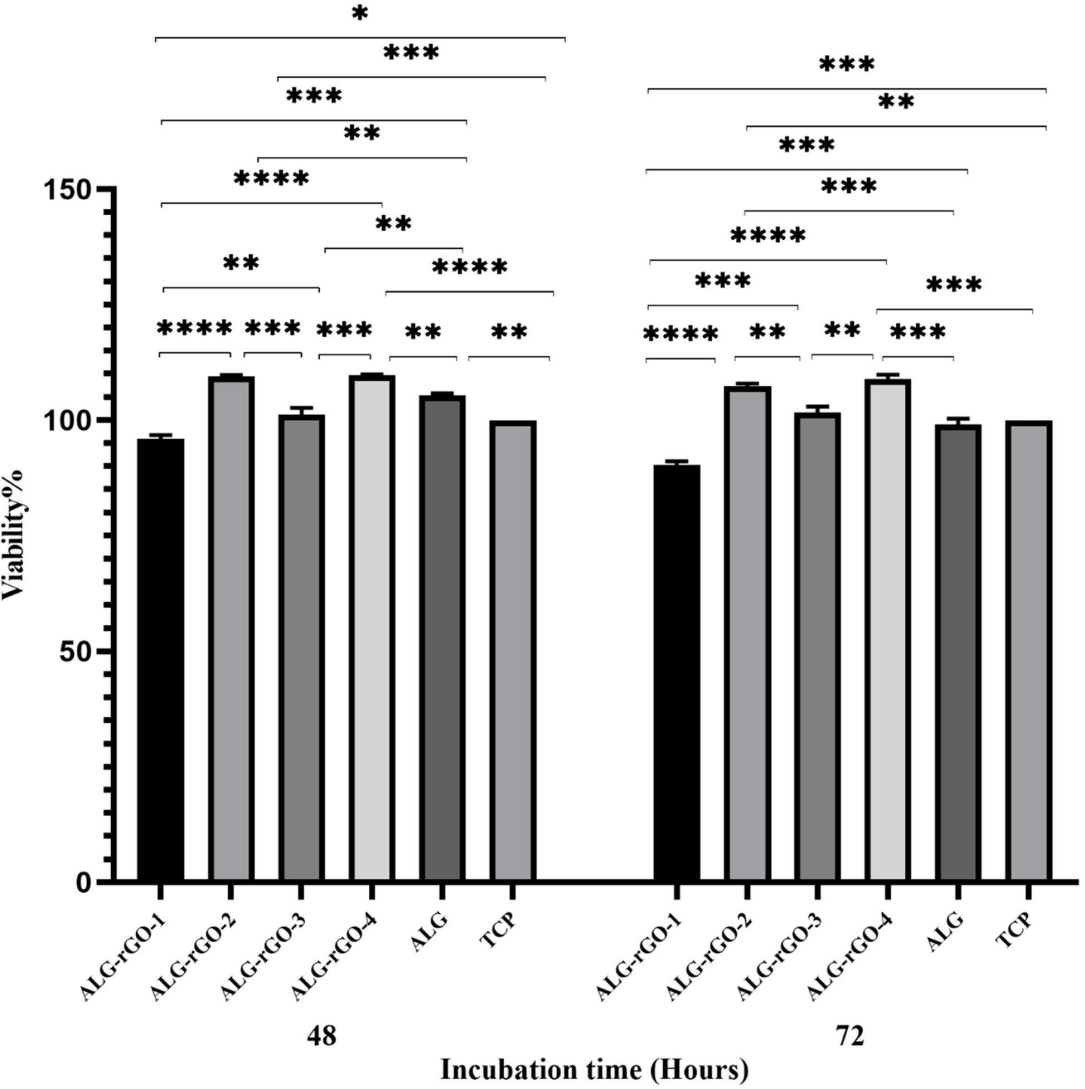
MTT assay results of prepared scaffolds on HUVECs viability. * (*p* < 0.05), ** (*p* < 0.01), *** (*p* < 0.001) and **** (*p* < 0.0001) indicate significant difference compared with other groups. Data were presented as the average ± standard deviation (*n* = 3)

The results of the cell viability test against HUVECs are presented in Fig. 8. Among coated scaffolds, ALG-rGO-2 and ALG-rGO-4 showed the highest viability (almost 110% ± 1.73), while ALG-rGO-1 demonstrated the least cell viability (nearly 95% ± 2.57) compared with that of other samples at both examined times. Improving cell viability showed no significant toxicity arising from rGO coating, as this value remained higher than about 95% for all samples at both studied time points.

**Fig. 9:**
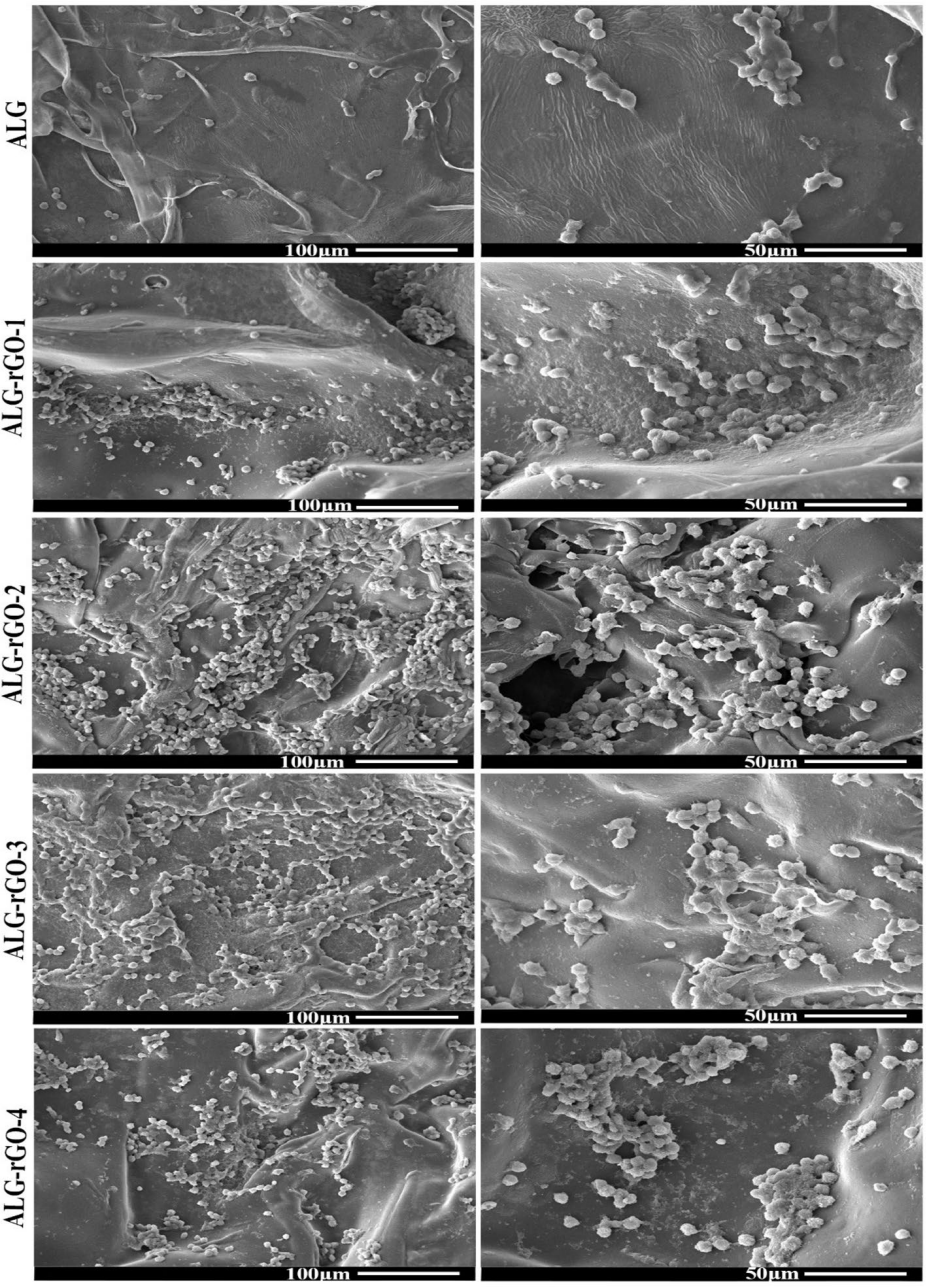
SEM micrograph of HUVECs seeded on scaffolds after 48 h with different magnifications

SEM micrographs of seeded HUVECs were taken at 48 h post-seeding. As shown in Fig. 9, all samples had cell attachment on their surface, which was enhanced by increasing the rGO concentration. This result indicates that the presence of rGO could facilitate and support cell adhesion and growth by providing a suitable substrate.

**Fig. 10:**
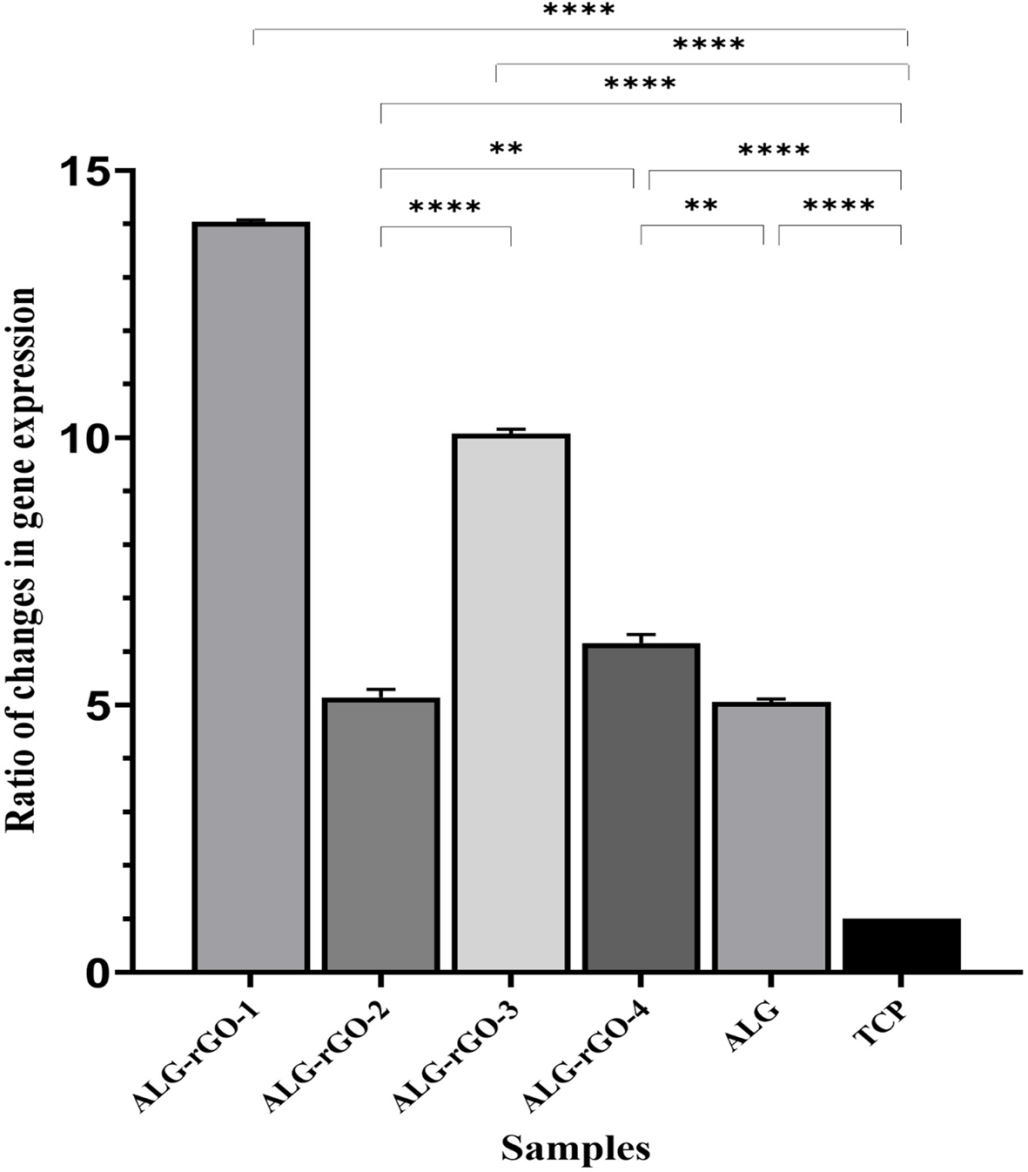
Gene expression of VEGFR2 by qRT-PCR analysis after 7 days of cell culturing on the scaffolds. * (*p* < 0.05), ** (*p* < 0.01), *** (*p* < 0.001) and **** (*p* < 0.0001) indicate significant difference compared with other groups (*n* = 3)

**Fig. 11:**
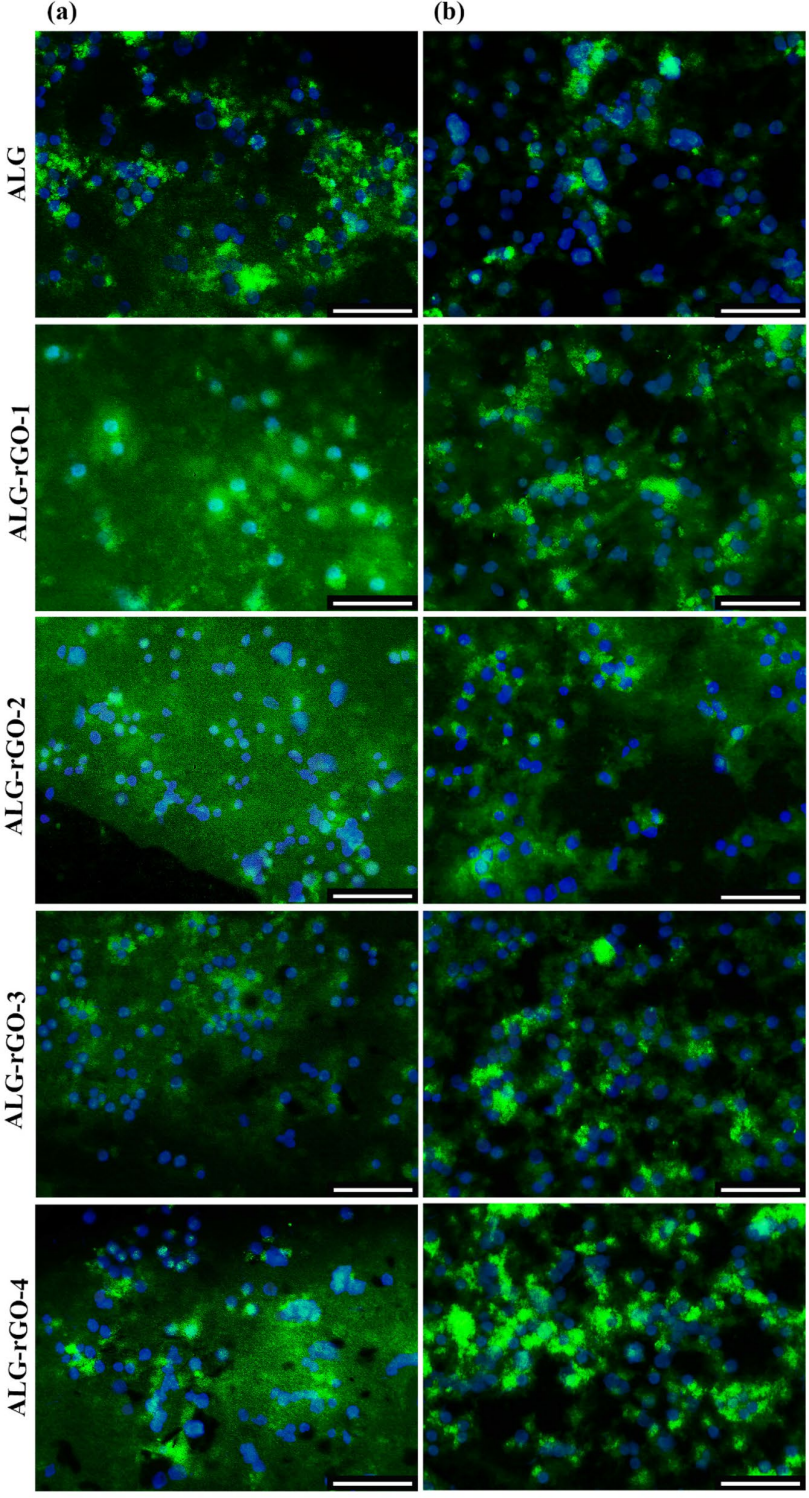
Immunofluorescence staining (blue: DAPI, green: VEGFR2) analysis for HUVECs after 7 (**a**) and 14 (**b**) days of HUVECs culturing on the scaffolds. Scale bar = 100 µm

**Fig. 12:**
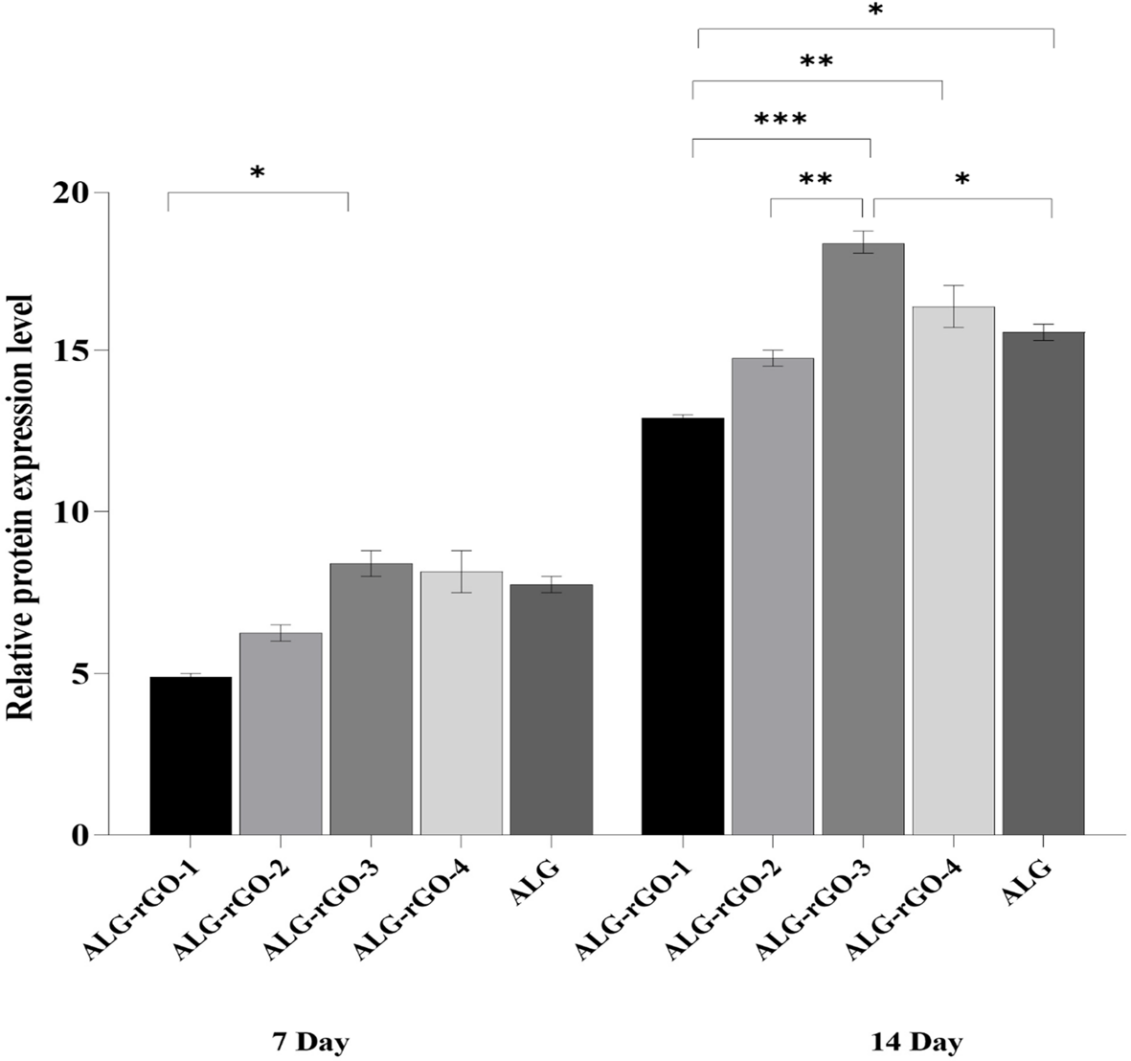
Quantitative analysis of protein expression of VEGFR2 after 7 and 14 days. * (*p* < 0.05), ** (*p* < 0.01) indicate significant difference compared with other groups

The expression profile of VEGFR2 for HUVECs seeded on the scaffolds was investigated by qRT-PCR and depicted in Fig. 10. The primer sequences and their annealing temperatures have been presented in Table 3. As can be seen, the corresponding gene expression was increased statistically in all electroactive scaffolds compared with ALG and TCP. This value was more significant in ALG-rGO-1 and ALG-rGO-3 at 14 and 10 folds, respectively (*p* < 0.0001). HUVECs grown on ALG also showed a fourfold increase in the level of VEGFR2 expression compared with that grown on TCP. For comparative evaluation of protein expression of VEGFR2 for HUVECs, immunofluorescence staining was conducted after 7- and 14-days post-seeding and quantified using Fiji/ImageJ. As shown in Fig. 11, protein expression of VEGFR2 was detected in all scaffolds, and was enhanced after 14 days. According to the calculated value after 7 days, although protein expression increased as the rGO coating concentration reached 0.3% w/v, it was not statistically significant compared to that of ALG. The same trend in which coating improved protein expression was observed among the prepared electroactive scaffolds following 14 days. However, in contrast to 7 days, the value was statistically different in ALG-rGO-3 compared to the ALG scaffold (*p* < 0.05) (Fig. 12).

**Table 3:** Primers used for qRT-PCR

Genes	Primer sequence (5′-3′)	Annealing (°C)
VEGFR2	F CGGTCAACAAAGTCGGGAGA R CAGTGCACCACAAAGACACG	60
GAPDH	F GACATGCCGCCTGGAGAAAC R AGCCCAGGATGCCCTTTAGT	60

### Antibacterial and antioxidant activity

**Fig. 13:**
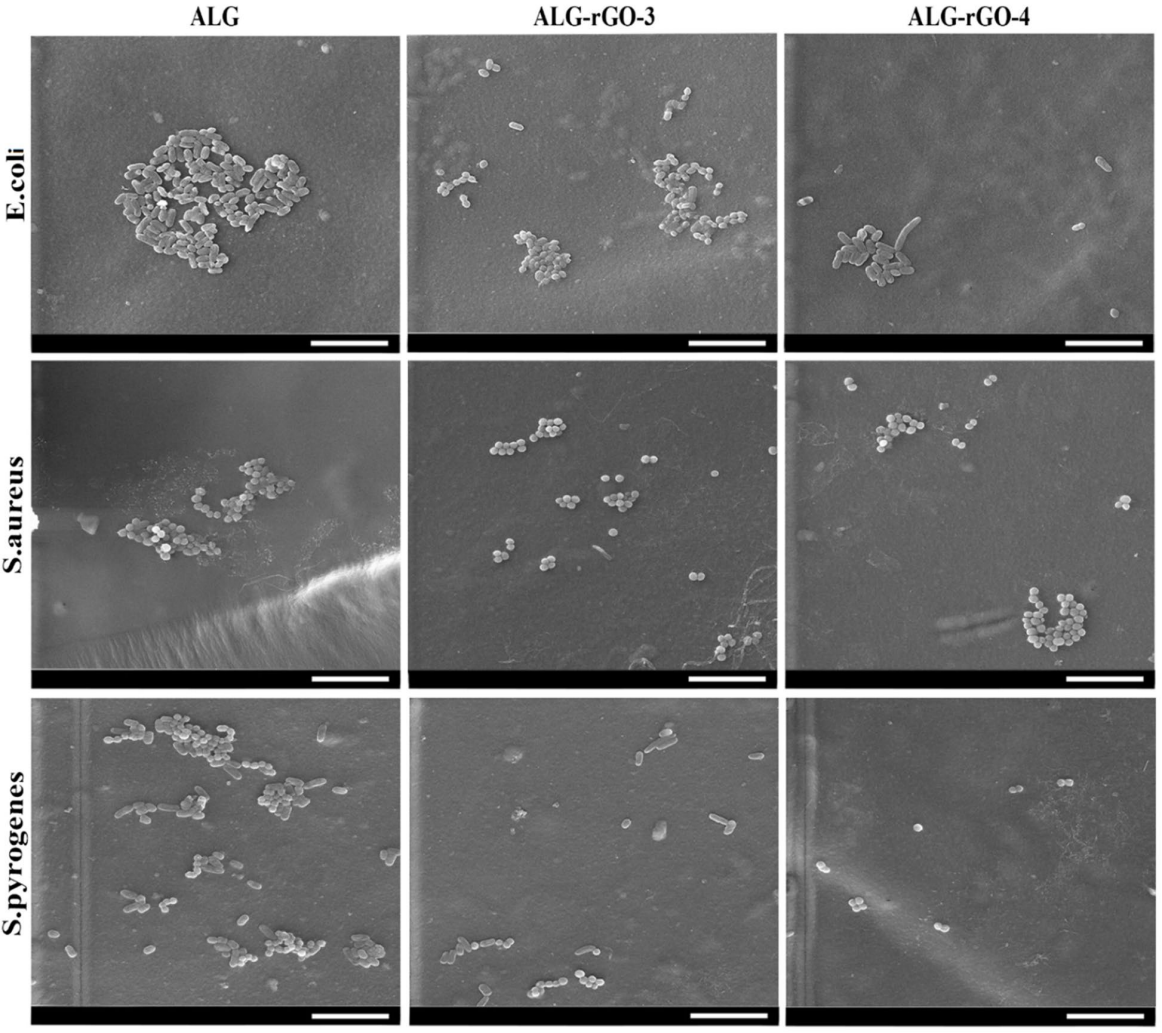
FE-SEM images of three species of bacteria attached to the prepared scaffolds. Scale bar = 10 µm

**Fig. 14:**
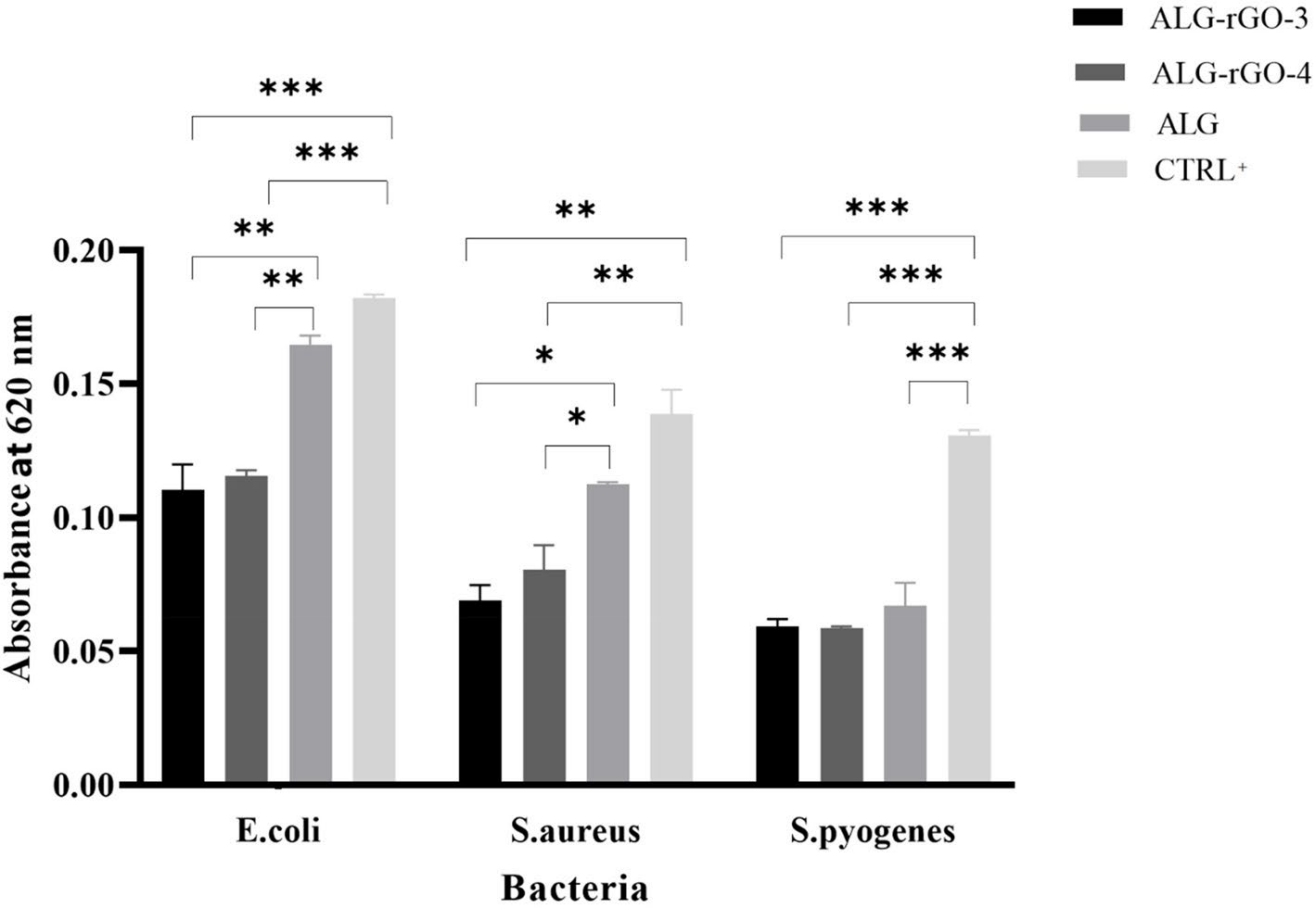
Growth rate of three species of bacteria in the presence of the scaffolds and the positive control (blank disk on MHA with each bacteria). * (*p* < 0.05), ** (*p* < 0.01) and *** (*p* < 0.001) indicate significant difference compared with other groups (*n* = 3)

The interaction between bacteria and the samples was evaluated by an antimicrobial test using *S. aureus*, *S. pyogenes,* and *E. coli* strains as representatives of Gram-negative and Gram-positive bacteria associated with endocarditis. Among prepared samples, ALG-rGO-3 and ALG-rGO-4, demonstrated a suitable contact angle for cell adhesion, higher conductivity compared with other samples, promising mechanical properties, a slower degradation rate, and higher VEGFR2 protein expression in HUVECs seeded on these scaffolds. As a result, they were selected as optimal samples for the following experiments, and subsequent tests were performed on ALG as uncoated and ALG-rGO-3, ALG-rGO-4 as electroactive scaffolds. The results of the FE-SEM evaluations demonstrated that while ALG showed considerable adhesion of the bacterial strains, the rGO-coated scaffolds effectively inhibited the attachment of bacteria onto their surface. Among the three examined bacterial strains, the growth of *S. pyrogene* was more inhibited by coated scaffolds, especially by ALG-rGO-4 (Fig. 13). The OD620 assay also demonstrated a significant difference in the growth rate of *S. aureus* (*p* < 0.05) and *E. coli bacteria* (*p* < 0.01) in the presence of rGO-coated scaffolds as opposed to those for ALG (Fig. 14). The inhibition rate of all bacteria was higher for ALG-rGO-4 among rGO-containing scaffolds, but not significantly higher than ALG-rGO-3 scaffold. Also, in line with the FE-SEM results, the growth of *S. pyrogene* was more suppressed, followed by *S. aureus* for all scaffolds.

**Fig. 15:**
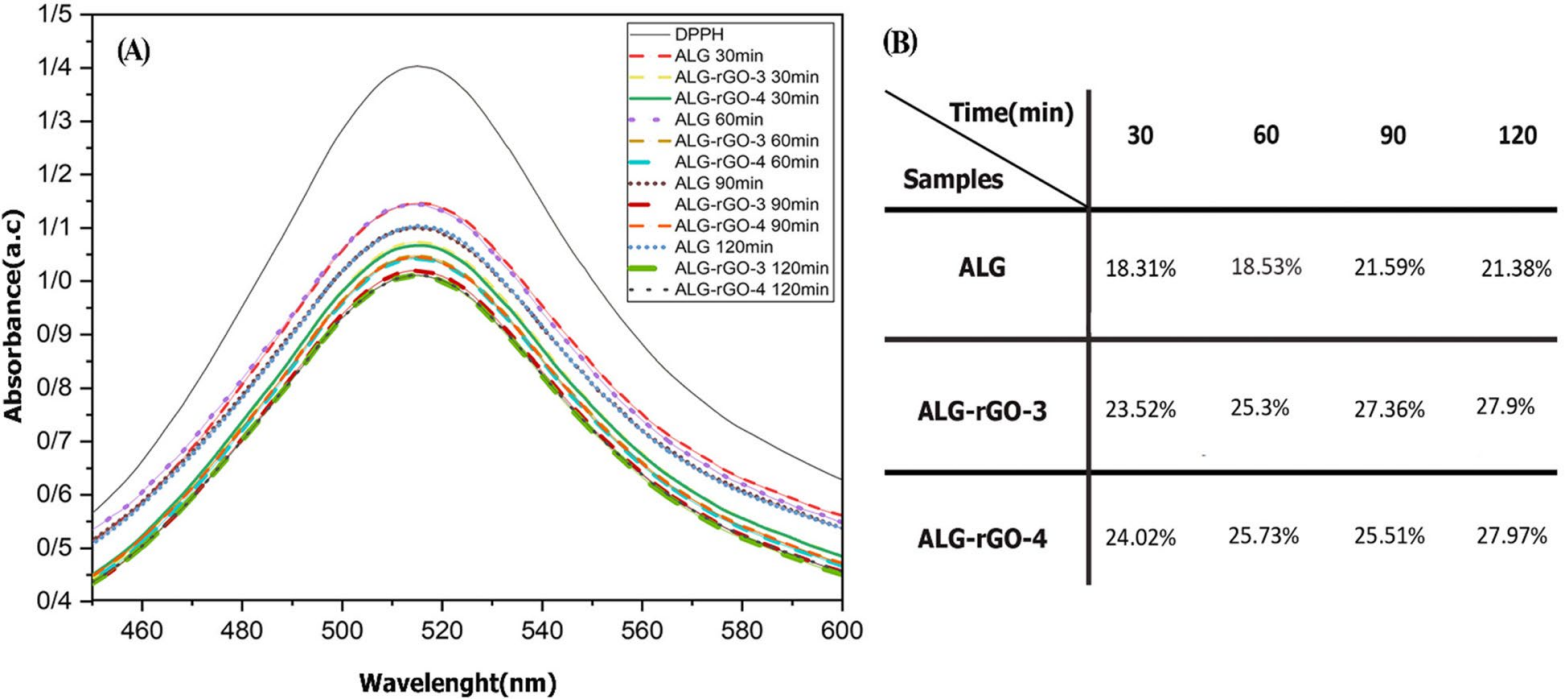
**A**) Antioxidant activity of the scaffolds using DPPH free radical scavenging assay, **B**) The calculated percentage of DPPH scavenging of the scaffolds over 120 min (*n* = 3)

In vitro antioxidant activity of coated scaffolds was measured by DPPH scavenging assay for 120 min, and the results were compared with those of ALG scaffolds. According to Fig. 15, the value was increased over the course of our experiment for all scaffolds, which was more noticeable in ALG-rGO-3. Interestingly, this scaffold exhibited improved anti-oxidizing ability at 28.01% compared to the ALG scaffold at 21.38% after 120 min.

### In vivo assessments and Immunohistochemical characteristics of CD31 and α-SMA

**Fig. 16:**
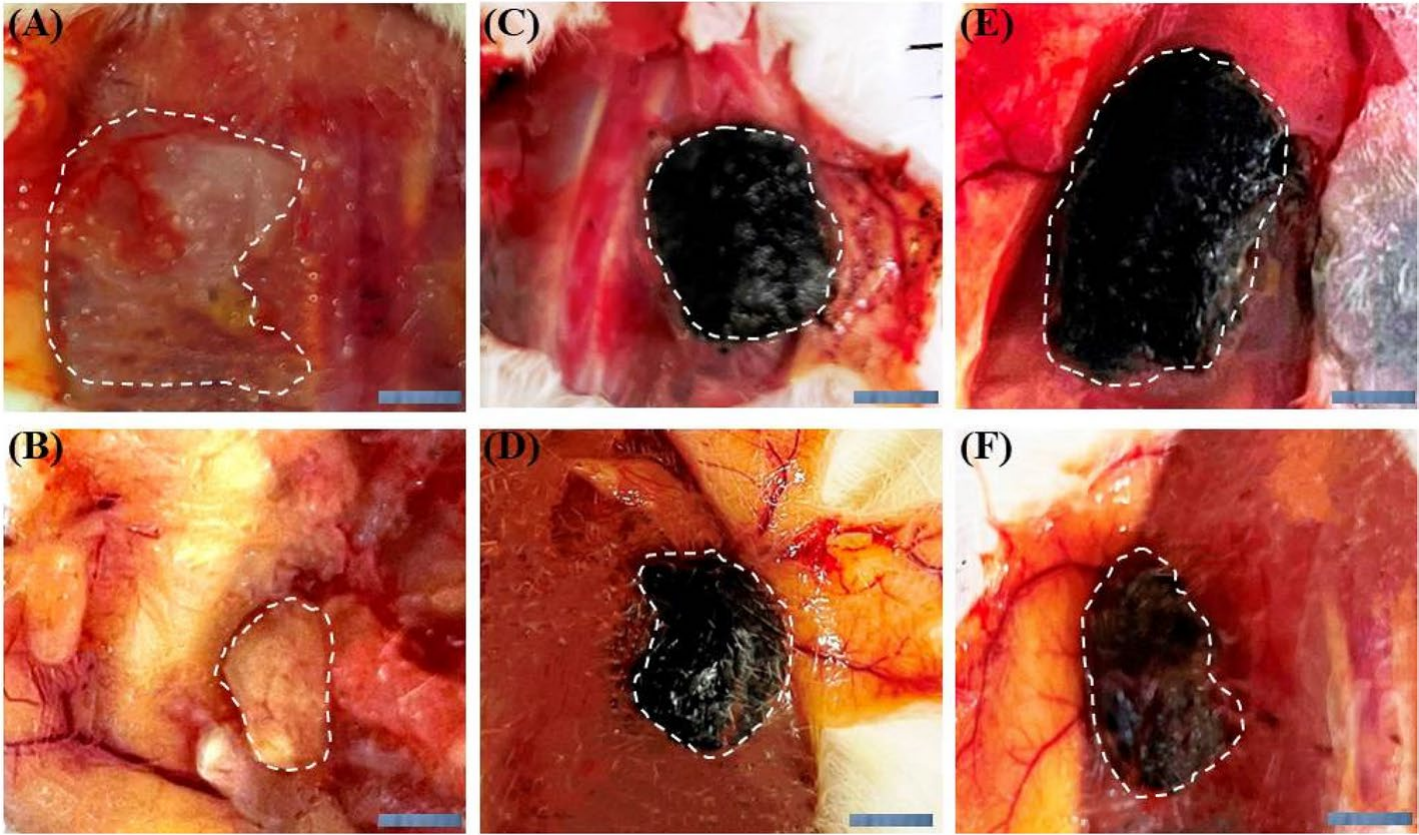
Gross morphology of the scaffolds after 1 (**A**, **C**, **E**) and 3 (**B**, **D**, **F**) weeks subcutaneous implantation. ALG (**A**, **B**) ALG-rGO-3 (**C**, **D**) ALG-rGO-4 (**E**, **F**). The dotted lines show the boundaries of the scaffolds

In vivo vascularization was evaluated by retrieving the subcutaneously implanted scaffolds after 1 and 3 weeks. Although the amount of vascularity observed around the scaffolds in all three groups appeared to be the same during the first week, more vessels were detected macroscopically around the electroactive scaffolds after 3 weeks. Obviously, scaffolds lost their defined morphology after 3 weeks, but degradation was more noticeable in ALG scaffolds (Fig. 16).

**Fig. 17:**
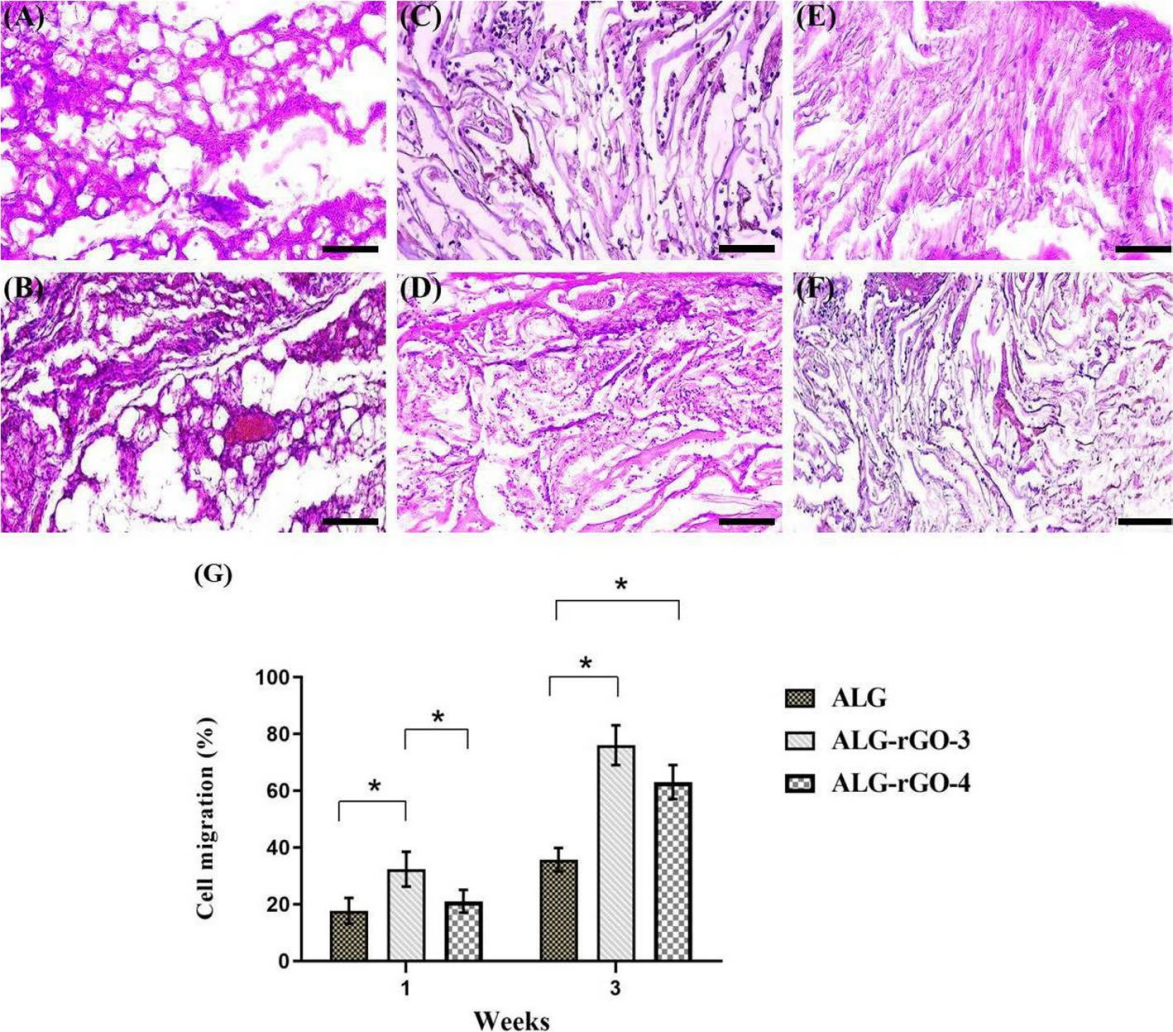
H & E staining of the scaffolds after 1 (**A**, **C**, **E**) and 3 (**B**, **D**, **F**) weeks subcutaneous implantation; ALG (**A**, **B**), ALG–rGO-3 (**C**, **D**), ALG-rGO-4 (**E**, **F**). Scale bar = 200 µm. Quantitative analysis of cell migration (**G**). * Specifies a statistically significant difference (*p* < 0.05)

The H&E images presented improved perivascular localization in the rGO-containing scaffolds compared with that of ALG. While three scaffolds became cellularized after 3 weeks (Fig. 17), the cell migration rate for electroactive scaffolds significantly surpassed that of ALG samples. After 3 weeks, the percentage of cell migration within ALG (35%) reached approximately 65 and 75% for ALG–rGO-4 and ALG-rGO-3, respectively which further shows the potential of rGO incorporation in promoting cell migration and tissue regeneration.

**Fig. 18:**
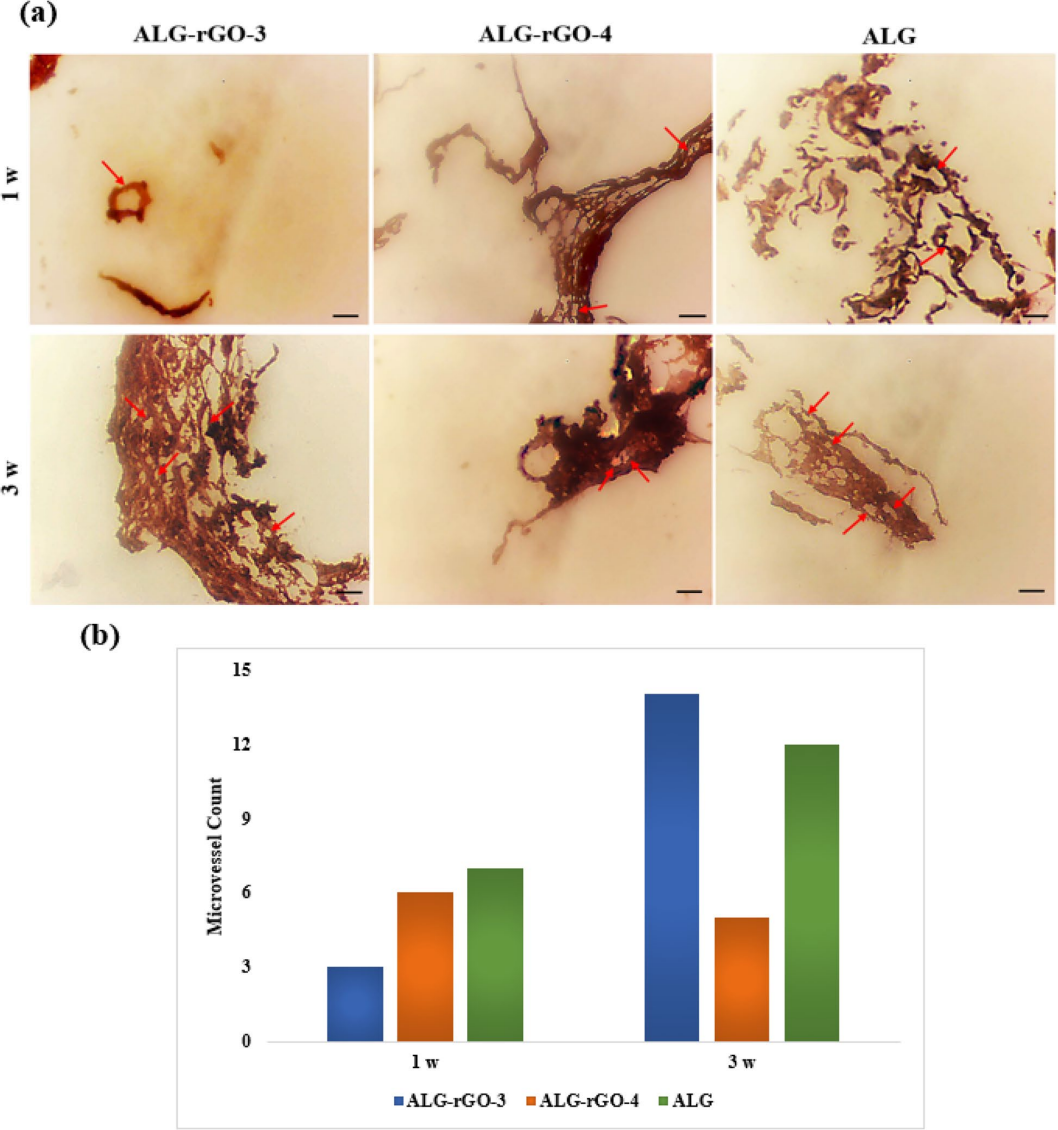
**a**) CD31 IHC expression compared in subcutaneously implanted scaffolds after 1 and 3 weeks. Arrows indicate endothelial cells (All panels 400 X). **b**) Quantified microvessels of the samples using image-J software

Immunohistochemical staining of tissue sections with CD31 and α-SMA was used for the study of the newly formed blood vessels. The total MVD in the scaffolds was determined using CD31 immunohistochemistry (Fig. 18a). While the electroactive scaffolds showed a lower CD31 expression compared with ALG in the first week, the value increased for ALG-rGO-3 after 3 weeks, the highest among the scaffolds (Fig. 18b).

**Fig. 19:**
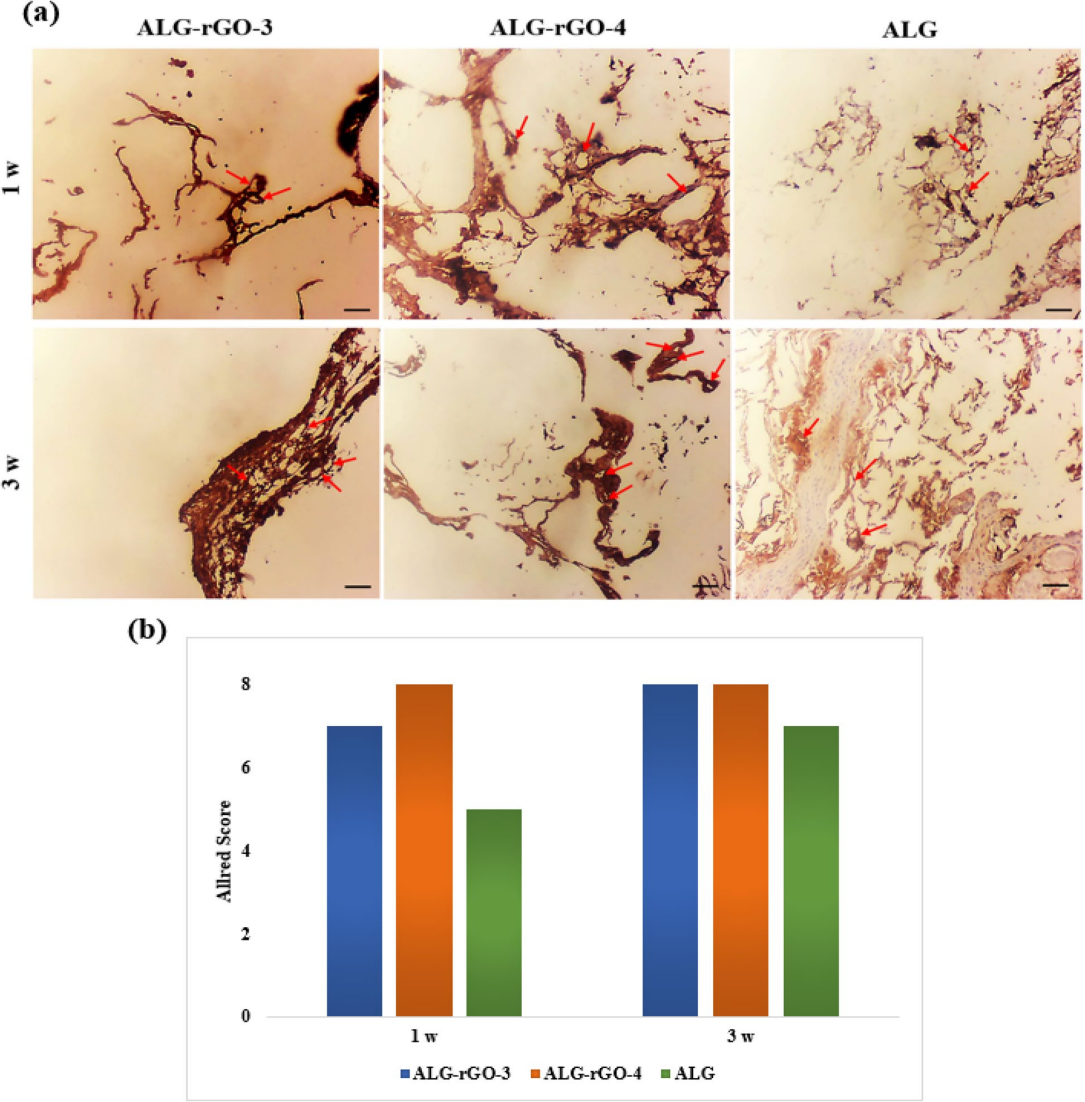
**a**) α-SMA IHC expression compared in subcutaneously implanted scaffolds after 1 and 3 weeks. Arrows indicate mural cells (All panels 400 X). **b**) Allred scoring of the samples utilizing image-J software

The Allred score (Table 4) was used to calculate the positively expressed α-SMA in epithelial cells (Fig. 19a). All samples were strongly positive for this marker indicating higher Proportion and Intensity scores after 3 weeks. However, the quantitative total score was higher for both GO-containing scaffolds at 8 compared with that in ALG scaffold at 7 (Fig. 19b). It should be pointed out that for both angiogenic markers, detected vascular lumens were considered positive by the pathologist, and the created background was ignored.

**Table 4:** Allred score^a^ for α-SMA evaluation

(Immunoreactivity of the cells%)	Proportion Score	Intensity	Intensity Score
0	0	None	0
< 1	1	Weak	1
1–10	2	Intermediate	2
11–33	3	Strong	3
34–66	4		
≥ 67	5		

^a^Allred score = Proportion score + Intensity score

## Discussion

Electroactive scaffolds are encouraging for cardiac tissue engineering by improving the synchronous contraction of CMs which is critical for efficient integration into the host tissue. Numerous studies have incorporated GBMs into polymeric scaffolds that could be applied as cardiac patches to enhance their mechanical properties and electrical conductivity [6, 53]. GBMs have significant mechanical and electrical qualities and the ability to interact with biological molecules, improving cell–cell signaling, cell propagation, and differentiation. Besides, their antibacterial properties can restore the functionality of the ischemic tissues [54]. Among GBMs, rGO seems more beneficial for cardiac tissue engineering, improving mechanical properties, electrical conductivity, and biocompatibility [55]. Our group has previously developed conductive scaffolds by chemically coating GO onto collagen (COL) scaffolds, followed by reduction of the oxygen-containing GO groups to create different concentrations (ranging between 5 and 90 μg/mL) of rGO coating. The rGO concentration was directly correlated with an increase in the scaffolds' mechanical and electrical conductivity. In addition, the expressions of the cardiac genes *Gja1, Actn4, and Tnnt2,* were unregulated compared to the collagen scaffold [12]. Later, we confirmed that rGO coating provides antibacterial properties over COL scaffolds to reduce the risk of infection related to cardiac patch implantation [41]. In the present study, we fabricated a series of ALG scaffolds via freeze drying, which were coated with higher concentrations of rGO (ranging between 5 and 30 mg/mL) for cardiac patch application.

SEM images showed that by increasing the rGO concentration, the pore size of the scaffolds decreased, which was well in agreement with the previous study [41]. Although ALG-rGO-3 and ALG-rGO-4 showed somewhat closed pore structures, the cross-section images showed that the porous interconnected structure of the scaffolds is still preserved after coating. Also, the acquired pore size for all scaffolds was still in an appropriate range for vascularization and survival of the cardiac tissue [56]. While pores smaller than 60 m are preferable for endothelial cell penetration and colonization for vascularization, larger pores allow nutrient diffusion throughout the scaffold [57]. Consistent with previous findings that rGO increased endothelial cell proliferation [58], the angiogenic potential of the rGO coating on our ALG substrate was also evident in the adhesion, spreading, and elongation of the HUVECs across all scaffolds, as seen in SEM images. The hydrophilicity of the scaffolds has a significant role in cell interactions. The water contact angle was reduced by increasing the rGO content. However, the obtained result remained in an appropriate range for cell adhesion. Investigations have shown that water contact angles in the range of 40–80 degrees provide the desired affinity for supporting different cell types [59, 60]. The obtained results were in line with our previous experiment, which demonstrated that rGO coating on the COL scaffold decreases the contact angle, confirming the substrate hydrophobicity [61]. The durability of cardiac patches depends on their biodegradability [62]. Coating on ALG scaffolds altered bulk properties and consequently modified the polymer's degradation kinetics. Aside from lowering the degradation rate after rGO addition, coated scaffolds showed a more gradual degradation trend than pristine ALG. The corresponding data support the contact angle result and demonstrate that the addition of rGO increased surface hydrophobicity, which in turn decreased degradation. Enzymatic degradation of ALG-rGO scaffolds will be studied in the future, particularly in the presence of lipase-type enzymes to better mimic physiological conditions than hydrolytic degradation [63–65]. Also, to understand rGO-ALG patch-host heart interactions, an assessment of in vivo degradation is necessary. In the future, our conductive rGO-ALG scaffolds can be combined or seeded with stem cells to accelerate angiogenesis, decrease inflammation, and activate CMs renewal, similar to previous research [62]. The electrical conductivity of rGO-coated ALG scaffolds obtained in the range of semiconductors (10^2^ –10^−6^ S/m) was appropriate for tissue engineering [12]. The high electrical conductivity of scaffolds could provide a suitable environment to improve cell–cell communications [66]. The conductive rGO networks in these scaffolds may deliver extra pathways for directing electrical current flow, decreasing the impedance for charge redistribution and action potential propagation [6].

One of the critical characteristics of cardiac tissue engineering is fabricating scaffolds with appropriate mechanical properties close to the native cardiac ECM. Cardiac patch stability after implantation on beating myocardium and assisting impaired myocardium from additional mechanical remodeling such as LV dilation are other essential features that should be precisely evaluated [15, 67, 68]. The incorporation of rGO enhanced the mechanical properties of pure ALG scaffolds to match the mechanical properties necessary for clinical applications. The results were in accordance with the previous study, in which the rGO coated COL scaffold provided about a three-fold increase in compressive strength. It has been stated that GO aggregation by the reducing process provides a tough coating over the scaffold due to the van der Waals forces between rGO monolayers, which is beneficial in improving mechanical properties [39]. In another study by Zhang et al., an electrospun scaffold comprised of silk fibroin and rGO reached a Young’s modulus of about 122.7 MPa, 9 folds higher than the pure silk fibroin. This increasing trend could be due to the thickening of the distributed GO layers after the reduction procedure [69]. Consequently, the existing gap between the layers is decreased, and the gravitational force of van der Waals is developed between the single layer of the GO coating, which would enhance the mechanical strength and Young’s modulus of the structure [70]. Also, Tri-layered electrospun ALG-GO/poly(*ε*-caprolactone) (PCL) has recently been fabricated as a cardiac patch. A combination of 0.5 wt% GO into the top and bottom alginate nanofibrous layers, increased the Young’s modulus of the prepared composite to 4.35 ± 0.56 MPa [71]. Because the human myocardium has an end-diastolic Young's modulus ranging between 0.2 and 0.5 MPa [72], the obtained results for the Young's modulus of the prepared scaffolds fell within this range. While the scaffolds' tensile strength was higher than that reported for native myocardium (40–200 kPa vs. 3-15 kPa), it has been demonstrated that cardiac patches with higher stiffness values (200% of the average stiffness of the passive myocardium) could benefit from better CMs function and reduced LV remodeling due to reduced wall stress without diastolic dysfunction onset [73].

The results of the MTT assay showed increased HUVECs viability for all samples compared with TCP after 48 h and 72 h. The physicochemical surface characteristics of rGO with fewer oxygen-containing groups and extra conjugated carbon structures than GO allow specific proteins from the culture media to be adsorbed on the rGO-coated scaffolds through hydrogen bonding, hydrophobic interactions, and electrostatic forces, which help cellular adhesion and organization [74]. However, GO and rGO could create cytotoxicity, oxidative stress, and DNA damage in mammalian cells in a dose-dependent manner. The fundamental mechanism of cell toxicity induced by graphene is its physical interaction with a cell membrane, in which the sharp edges of a graphene sheet may damage the cell membrane, resulting in intracellular content leakage [55]. Additionally, GO promotes nicotinamide adenine dinucleotide phosphate oxidase-dependent ROS formation coupled with the deregulation of antioxidant genes, whereas rGO induces physical stress resulting in increased ROS production [75]. Sasidharan et al. demonstrated that few-layer graphene (~ 160 ± 48.5 nm) could cause oxidative stress in HUVECs due to changes in the plasma membrane integrity, decreased metabolic activity, high levels of ROS production, depolarization of mitochondrial membrane potential, and lipid peroxidation stimulation. However, the authors stated that cell toxicity could be altered depending on the surface chemistry, including hydrophilicity, the multi-layered nature of the graphene, and the differences in various sources of cells [76]. Our group also previously developed conductive scaffolds by chemically coating rGO onto COL scaffolds with different concentrations. In that experiment, we observed a trend in which increasing the rGO concentration from 400 to 600 and 800 μg/ml decreased HUVECs viability [41]. While the concentrations used to coat the ALG in the present study were higher than those used for COL in our aforementioned study, higher cell viability was observed by increasing the rGO concentration to 0.3% w/v. Therefore, apart from the concentration, the polymer type on which GO or rGO is coated (ALG vs. COL) and the type of coating (physical vs. chemical) could influence the toxicity of the scaffolds, affecting cell viability.

Angiogenesis plays a significant role in treating cancer, atherosclerosis, wound healing, and cardiovascular diseases [77]. The absence of angiogenesis is a critical problem in the recovery post-MI, which leads to the permanent death of CMs in the first 2–4 h of the onset [78]. To address this deficiency in neovascularization, rGO could adsorb ECM proteins such as fibronectin from the cell culture medium containing serum, which could subsequently improve cell-ECM interactions through FN-integrin binding and enhance expressions of phosphorylation of focal adhesion kinase (pFAK), phosphorylated extracellular signal-regulated kinase (pERK), and thus VEGF which is demanding for cardiac repair [22]. The promotion of angiogenesis could reduce cardiac remodeling after MI and increase the regeneration rate of damaged heart tissue [79]. In this study, we evaluated the angiogenic properties of the rGO coating after 7 days of incubation with HUVECs. Although all electroactive samples presented a significantly higher VEGFR2 expression compared with TCP and ALG, this value was more noticeable for ALG-rGO-1. When HUVECs are exposed to ALG-rGO scaffolds, their angiogenic potential can therefore be boosted. Our findings demonstrating an upregulation of VEGFR2 expression on rGO-coated scaffolds provided further evidence for this hypothesis. However, assessment of other angiogenic factors in vitro and the tube formation assay are required to find a rational relationship between the concentration of rGO and the level of angiogenic factor expression (for both the gene and protein). Also, if we had continued the gene expression analysis for 14 days, we might have discovered a rational relationship between the increase in rGO concentration and gene expression.Immunofluorescence staining performed 7 and 14 days after HUVECs seeding revealed that electroactive scaffolds had higher protein expression compared to ALG. Among coated scaffolds, ALG-rGO-3 performed best at both time points. Moreover, the obtained results on the angiogenic capability of the electroactive scaffolds were further confirmed following the retrieval of the subcutaneously implanted scaffolds. Over the course of three weeks, the rate of cell migration within rGO-ALG scaffolds was significantly higher than that of ALG, with the difference being most pronounced for ALG-rGO-3. The angiogenic capacity of the scaffolds was also assessed by IHC against CD31 and α-SMA markers. Our results confirmed the potential of rGO-coated scaffolds, which further prove that they can stimulate neovascularization in developing tissues. Considering both markers, ALG-rGO-3 showed higher microvessels with a strong Allred score for α-SMA. However, there is a limitation to this assessment for staining the background, which could be due to the reaction of the scaffolds with the corresponding stain. For this reason, only lumens containing endothelial cells were counted by the pathologist. Previous study has shown that both GO and rGO could be pro-angiogenic. It was stated that ROS could induce Akt phosphorylation and activate the nitric oxide (NO) signaling pathway by stimulating endothelial NO synthase (eNOS) (upregulation of phospho-eNOS). Improvement of intracellular NO production finally activates angiogenesis [77]. We have also previously confirmed that carbon-based materials, including rGO and CNTs, could improve the angiogenic properties of the scaffolds effectively [12, 27, 80]. In vivo  assessment of subcutaneously implanted rGO-COL scaffolds after 1 and 3 weeks post-implantation in mice further approved improved cell infiltration within the electroactive scaffolds as well as the angiogenic properties of rGO-incorporated constructs due to the presence of rGO [12]. Altogether, the increase in gene and protein expression of VEGFR2 on the rGO-coated scaffolds could be due to the angiogenic-inducing properties of rGO, which have been similarly shown in previous studies [81, 82].

Infective endocarditis (IE) occurs after the implantation of cardiovascular devices, including prosthetic valves, implantable cardioverter-defibrillators, and pacemakers [83]. Among specific organisms, *S. aureus* is the primary reason for acute IE, becoming a major cause in most records [83]. Additionally, even though rare, *E. coli* and *S. pyogenes* have been seen as infectious etiologies and the cause of endocarditis, especially in patients who received prosthetic mitral valve [84]. The antibacterial activities of GBNs might be due to either an oxidative stress effect or membrane disruption [85]. Damage to the bacterial membrane integrity caused by direct interaction with graphene sheets and subsequent generation of oxidative stress could be considered as reasons for bacterial eradication, depending on the bacteria type,and the physicochemical features of graphene materials, including functional group density, conductivity, concentration, nanosheet size, and exposure time [29, 86]. Previously, we confirmed the antibacterial activity of the prepared COL cardiac patch containing rGO against *S. aureus*, *E. coli*, and *S. pyogenes* [41]. Herein, we effectively observed the excellent antibacterial activity of the prepared scaffolds against the three aforementioned bacterial strains, which could be advantageous for inhibiting IE for future clinical applications. Among the examined bacterial strains, the growth of *S. pyrogene*, while not significant, was more suppressed by ALG-rGO-4 with a concentration of 0.3% w/v coated rGO followed by *S. aureus*. Although inhibition of both bacteria strains was seen, rGO could more effectively suppress the gram-positive bacteria than the gram-negative ones. Similar observations were stated in previous studies, in which *S. aureus* cells had higher susceptibility to GO sheets than *E. coli* [87]. However, our findings contradicted previous research, which found that adding rGO to isabgol nanocomposite scaffolds inhibited *E.coli* colony formation more than *S.aureus* [42]. There are still conflicting reports on the antibacterial activity of GO and rGO. For example, previous studies showed that GO lacks antibacterial effects and may act as a scaffold for the attachment and proliferation of bacteria and biofilm formation [88]. In another study, it was stated that GO sheets intertwine the bacterial spores, contributing to local perturbation of their cell membrane and reducing the potential of the bacterial membrane. All of which result in cell lysis and death [89]. Thus, more in-depth characterizations of the antimicrobial properties of GO and rGO are required to address the aforementioned discrepancies.

The pathological development of MI is significantly influenced by reactive oxygen species (ROS). MI tissue has a low antioxidant capacity and is vulnerable to ROS-induced damage. As a result, the potential for antioxidant therapy for MI is high [90]. However, there has been limited success with antioxidant therapy in the treatment of MI, and no antioxidants are currently approved for clinical use [90]. In this experiment, we evaluated the antioxidant capability of the prepared scaffolds to find out the possible inductive effect of rGO coating over ALG. Oxidation protection by carbon-based materials may include the formation of radical adducts on the surface of sp^2^ carbon that provides electrons to eliminate the free radicals [35]. In this study, the higher antioxidizing activity of rGO could result from the sp2 carbon reconstruction of GO by reduction, as previously reported [91]. Again, similar to the antibacterial and angiogenic evaluations, the ALG-rGO-3 scaffold outperformed the ALG scaffold in terms of anti-oxidizing properties in a DPPH scavenging assay, indicating that this is the scaffold for which the optimal dosage of the applied coating has been achieved. Consequently, rGO coating may lessen the damage caused by ROS in MI tissues, where a high amount of ROS accumulates in the infarcted region. In the future, we plan to examine the inflammatory response of our ALG-rGO scaffolds in a mouse model of MI using immunohistochemical staining of related inflammatory markers. Conclusively, rGO coating improved the physicochemical properties of ALG, which could provide a desired electroactive cardiac patch for ischemic heart disease patients. However, apart from the numerous experiments confirming the assuring characteristics of GBNs for cardiac tissue regeneration, many challenging questions still need to be addressed. Cytotoxicity, genotoxicity, and the biocompatibility of GBNs, which highly rely on dosage, exposure time, number of layers, lateral dimensions, shapes, and chemistry, should undergo extensive safety evaluations before considering these materials for clinical use. Besides, the elimination route of graphene from the body is another concern that has yet to be studied. We are aware that most cardiac patches require open-chest surgery, which increases complications and limits their therapeutic potential for clinical use. For this reason, minimally invasive delivery of cardiac patches is more desirable. Shape memory materials with stronger mechanical properties will be the next generation of biomaterials to be used for cardiac patch fabrication [92]. Apart from the above-mentioned questions, the findings of this study have to be seen in light of some limitations. Since rGO often experiences severe aggregation, particularly in hydrophilic or aqueous environments, which reduces its stability and efficacy for biomedical applications [91], scaffolds are unlikely to experience homogeneous coating using the stated method in this experiment. Physical coating, while simple and practical, was not reproducible enough because it relied heavily on manual sampling. Homogeneous distribution of the coating and the effect of the washing to finalize the coating process were other factors affecting the work outcome. For this reason, other coating methods that provide more control over the process are suggested. Further work is necessary to assess the biological characteristics of the prepared samples using cardiac cells, as well. In vivo assessment in a model of MI is also required to approve the capability of the prepared electroactive patches to stop the progression of the remodeling process. Despite the limitations mentioned earlier, the development of cardiac patches still breaks ground for cardiac repair and gives insight into heart regeneration.

## Conclusion

In this study, different concentrations of rGO were coated onto ALG scaffolds to create a series of conductive scaffolds for cardiac tissue engineering. The Young modulus and tensile strength of the scaffolds were improved by 187.33% and 81.81% when the concentration of rGO was raised from 0.05% to 0.3% w/v. The coated scaffolds' calculated electrical conductivity was within the range of semi-conductive materials (~ 10^−4^ S/m), making them suitable for cardiac tissue engineering. The upregulation of VEGFR2 expression confirmed that rGO coating significantly support the angiogenic capability of ALG against HUVECs. The antibacterial potential of ALG scaffolds was also improved by the rGO coating, making them more effective against *E. coli, S. aureus,* and *S. pyogenes*. In vivo evaluations also confirmed the enhanced vascularization properties of coated samples after subcutaneous implantation of uncoated and rGO-coated ALG scaffolds in mice. In conclusion, the findings suggest that rGO has the potential to provide ALG scaffolds with the necessary mechanical, antioxidant, antibacterial, and angiogenic properties, which play an essential role, particularly post-infarction.

## Data Availability

The datasets used and/or analyzed during the current study are available from the corresponding author upon reasonable request.

## Abbreviations

ALG: Alginate
GO: Graphene Oxide
rGO: Reduced Graphene Oxide
HUVECs: Human Umbilical Vein Endothelial Cells
CVD: Cardiovascular disease
MI: Myocardial Infarction
LV: Left Ventricular
CMs: Cardiomyocytes
HF: Heart Failure
GBMs: Graphene-based Materials
ROS: Reactive Oxygen Species
DMSO: Dimethyl Sulfoxide
VEGFR2: Vascular Endothelial Growth Factor Receptor 2
TCP: Tissue Culture Plate
MSCs: Mesenchymal Stem Cells
E. coli: Escherichia coli
S. aureus: Staphylococcus aureus
S.pyogenes: Streptococcus pyogenes
ECM: Extracellular Matrix
FTIR: Fourier transform infrared
AFM: Atomic Force Microscopy
XRD: X-ray diffractometer
ATR-FTIR: Attenuated Total Reflection-Fourier Transform Infrared Spectroscopy
SEM: Scanning Electron Microscopy
qRT-PCR: Quantitative reverse transcription PCR
pFAK: Phosphorylation of focal adhesion kinase
Perk: Phosphorylated extracellular signal-regulated kinase
COL: Collagen
